# Last glacial hydroclimate variability in the Yucatán Peninsula not just driven by ITCZ shifts

**DOI:** 10.1038/s41598-023-40108-6

**Published:** 2023-09-01

**Authors:** Leah Travis-Taylor, Martín Medina-Elizalde, Ambarish V. Karmalkar, Josué Polanco-Martinez, Gabriela Serrato Marks, Stephen Burns, Fernanda Lases-Hernández, David McGee

**Affiliations:** 1 Department of Earth, Geographic, and Climate Sciences, UMass Amherst, Amherst, MA USA; 2 Department of Geosciences, University of Rhode Island, Kingston, RI USA; 3 GECOS-IME, Campus Miguel Unamuno, Edificio FES, Salamanca, and Basque Centre for Climate Change (BC3), University of Salamanca, Leioa, Spain; 4 Department of Earth, Atmospheric, and Planetary Sciences, MIT, Cambridge, MA USA; 5 Centro de Geociencias, UNAM Campus, Blvd. Juriquilla 3001, 76230 Juriquilla, Mexico

**Keywords:** Climate sciences, Palaeoclimate, Hydrology

## Abstract

We reconstructed hydroclimate variability in the Yucatán Peninsula (YP) based on stalagmite oxygen and carbon isotope records from a well-studied cave system located in the northeastern YP, a region strongly influenced by Caribbean climate dynamics. The new stalagmite isotopic records span the time interval between 43 and 26.6 ka BP, extending a previously published record from the same cave system covering the interval between 26.5 and 23.2 ka BP. Stalagmite stable isotope records show dominant decadal and multidecadal variability, and weaker variability on millennial timescales. These records suggest significant precipitation declines in the broader Caribbean region during Heinrich events 4 and 3 of ice-rafted discharge into the North Atlantic, in agreement with the antiphase pattern of precipitation variability across the equator suggested by previous studies. On millennial timescales, the stalagmite isotope records do not show the distinctive saw-tooth pattern of climate variability observed in Greenland during Dansgaard–Oeschger (DO) events, but a pattern similar to North Atlantic sea surface temperature (SST) variability. We propose that shifts in the mean position of the Intertropical Convergence Zone (ITCZ), per se, are not the dominant driver of last glacial hydroclimate variability in the YP on millennial timescales but instead that North Atlantic SSTs played a dominant role. Our results support a negative climate feedback mechanism whereby large low latitude precipitation deficits resulting from AMOC slowdown would lead to elevated salinity in the Caribbean and ultimately help reactivate AMOC and Caribbean precipitation. However, because of the unique drivers of future climate in the region, predicted twenty-first century YP precipitation reductions are unlikely to be modulated by this negative feedback mechanism.

## Introduction

Dansgaard–Oeschger (DO) events represent abrupt climate shifts from relatively cold (“stadial”) to warm (“interstadial”) atmospheric conditions over Greenland^1,2^. Climate transitions were abrupt (gradual) from stadial (interstadial) to interstadial (stadial) conditions, producing a characteristic sawtooth shape. DO-resembling climate variability has been observed in marine and terrestrial records across the Northern Hemisphere e.g.,^3–5^. Particularly cold stadial intervals were accompanied by events of massive iceberg and freshwater discharge from Greenland into the North Atlantic, known as Heinrich (H) events^6,7^. When H events took place, the Atlantic Meridional Overturning Circulation (AMOC) weakened and/or shutdown due to salinity-driven density changes in regions of North Atlantic Deep Water (NADW) formation^8,9^. AMOC reduction/shutdown reduced heat transport from the Southern Hemisphere (SH) into the Northern Hemisphere (NH)^8–10^, ultimately influencing low latitude climate, including reductions in North Atlantic tropical and subtropical precipitation e.g.,^5,11–14^. Rainfall was impacted also in the SH during DO and H events, with hydrological variability that was out-of-phase relative to the observed pattern in the NH, as suggested by available paleo-rainfall records e.g.,^15–17^.

In tropical and subtropical regions, stalagmite deposits can represent archives of past hydroclimate information because their carbon (δ^13^C) and oxygen (δ^18^O) isotope composition may relate to precipitation amount; in the case of δ^13^C primarily via changes in prior calcite precipitation (PCP), degassing, bedrock dissolution, and soil productivity^18,19^, and in the case of δ^18^O via the *amount effect*^20,21^. There are currently few paleo-hydroclimate records from the Gulf of Mexico and Caribbean regions to establish the character of climate variability from decadal to millennial timescales during the last glacial interval. The few available paleoclimate records from the northern Caribbean region, for instance from Cuba, Puerto Rico, and Guatemala, are not all consistent with each other but generally show climate variability resembling DO cycles^11,13,14,22–24^. Lack of sufficient understanding of long-term hydroclimate dynamics in the broader Caribbean region hinders complementing model predictions of future precipitation change with empirical data, in a manner analogous to paleotemperature sensitivity studies^25^.

The Yucatán Peninsula (YP), Mexico, lies between the Caribbean Sea and the Gulf of Mexico and within a transitional zone between the tropics and subtropics^26^. Because YP’s climate regimes are highly influenced by Caribbean climate dynamics, there is a significant correlation between interannual precipitation variability in the YP and the broader Caribbean region^11,21,27^. In this study we present oxygen and carbon isotopic records from the YP that span the time interval between 43 thousand years (ka) before present (BP) and 26.6 ka BP, from a stalagmite named Sac Nicté (SN; “white flower” in Maya), after the legendary Maya princess of the ancient city Mayapán. The SN stalagmite was retrieved from one of the most thoroughly monitored cave sites in the northern tropics available to date^28,29^, which has yielded three speleothem paleoclimate records spanning the Holocene and last glacial^11,19,30^. The SN stalagmite thus covers an ideal time frame to investigate rapid climate change in the northern Caribbean region at the time when abrupt DO oscillations were taking place in Greenland^1,2^. Our new high-resolution speleothem stable isotope records enable examination of: (1) the frequency and character of hydroclimate variability in the northern Caribbean region from decadal to millennial timescales; (2) the antiphase precipitation pattern suggested by speleothem records across the equator; and (3) a potential negative climate feedback mechanism between Caribbean hydroclimate and AMOC.

### Study location and local climatology

**Figure 1:**
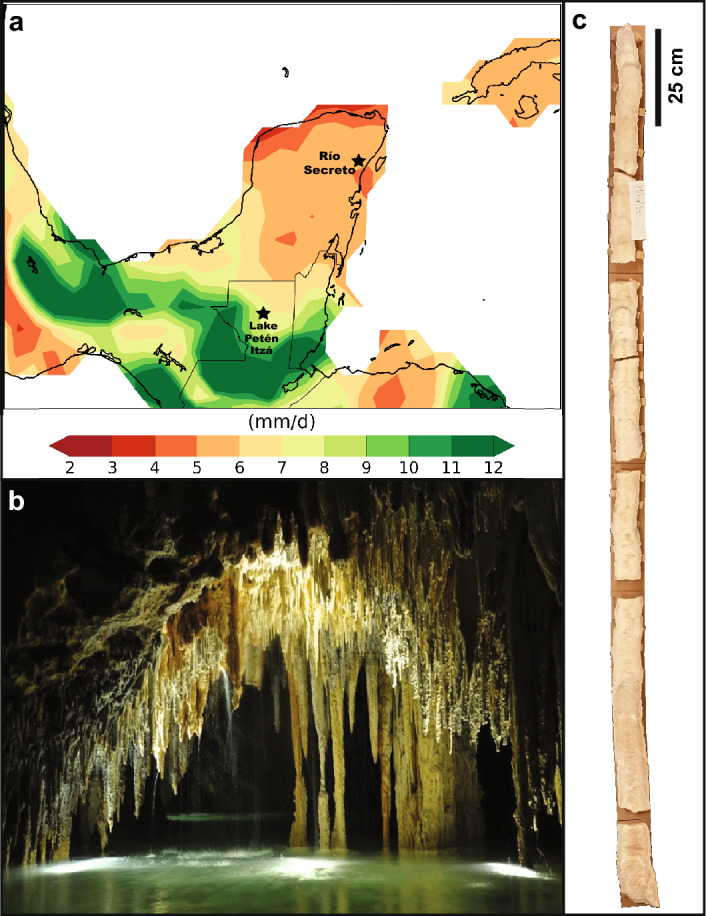
(**A**) Map of Yucatán Peninsula showing the location of Río Secreto cave (20° 35.244′ N; 87° 8.042′ W) and Lake Petén Itzá (16° 55′ N; 89° 50′ W). Shading represents rainy season (June-Oct) precipitation amount (mm/day) using the GPCC observational dataset^73^ (1981–2015) and illustrates the hydroclimate transition from subtropical (northwest) to tropical (southwest) conditions. Map generated in Python using Matplotlib (v. 3.3.4) and Iris (v. 3.1.0). (**B**) Picture of Río Secreto cave. (**C**) The 2-m-long Sac Nicté stalagmite specimen.

**Figure 2:**
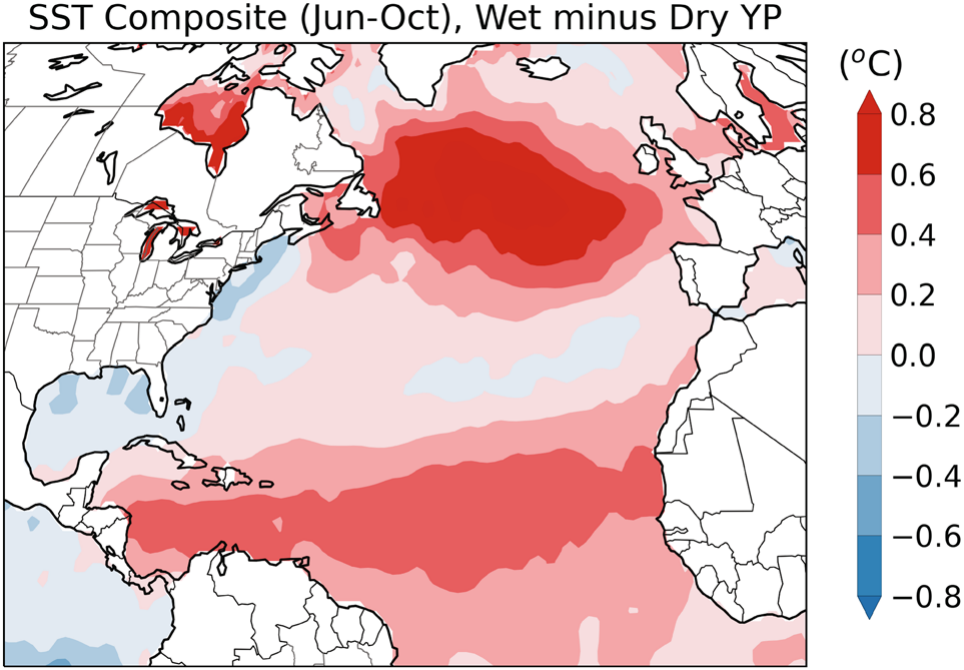
Plot of the difference between sea surface temperature (SST) composites, based on data for the period 1901–2019, for wet and dry years in the YP. Both precipitation and SST data were analyzed for the rainy season (Jun-Oct). Years for wet (9 years) and dry (7 years) composites were identified as years with precipitation in YP above and below 1.5 standard deviations, respectively, based on data between 1901 and 2019. The datasets used include Global Precipitation Climatology Centre (GPCC) data^73^ for precipitation and Hadley Centre Sea Ice and Sea Surface Temperature (HadISST) data^74^ for SST. Precipitation over the YP was calculated for the following domain: 87°–90.5° W, 18°–21.5° N. Figure generated in Python using Matplotlib (v. 3.3.4) and Iris (v. 3.1.0).

The YP has a latitudinal extent of ~4° (~17–21° N) and experiences three climatological seasons: *Rainy*, *Dry*, and *Nortes* (Fig. 1)^26^. The *Rainy* season, also known as the “hurricane” season, occurs between the months of June and October and dominates the annual rainfall budget with 50–90% of total annual precipitation falling during this time^29,31^. The *Dry* season (March–May) is characterized by high evaporation due to a more permanent influence of the North Atlantic subtropical high-pressure system, which produces low or no precipitation in the region. The *Nortes* season (November to February) brings the increasing influence of northwestern cold fronts, which lower atmospheric temperature and occasionally produce significant rainfall in the region^29^. A sharp precipitation gradient is observed both latitudinally and meridionally across the YP (Fig. 1). For example, at ~ 21° N the west receives < 500 mm/year while the east more than 1200 mm/year. Precipitation amount also increases from the northwest to the southeast, with ~ 2000 mm/year of precipitation in the southeast^26^. Although maximum precipitation in the YP occurs in the summer months when the ITCZ is located near its northernmost position in the North Atlantic, the center of ITCZ convective activity does not reach the latitudes of the YP^24,31,32^. This is one of the reasons why studies that investigate the relationship between large scale climate dynamics and precipitation regimes in the Americas fail to find a clear relationship between the area of influence of the ITCZ and precipitation regimes in the YP^33–35^. Seasonal precipitation variability in the YP is thus indirectly influenced by the movement of the ITCZ with other determining factors playing important roles, such as the strength and position of the subtropical high pressure system^27^, the Caribbean Low Level Jet (CLLJ)^34^, easterly waves, and sea surface temperatures (SST) in the Caribbean and tropical Atlantic^26,27,36^ and the eastern Pacific^37^. The dominant moisture source to the region is the North Atlantic and the Caribbean in particular^21^. Climate model projections indicate drier conditions in the YP in the future due to the intensification and western movement of the subtropical high pressure system, the strengthening of CLLJ, and the associated moisture divergence in the region^38^. These local factors that control YP hydroclimate over the instrumental period are shown to be related to North Atlantic-wide climate variability determined by Atlantic Multidecadal Variability (AMV), and on longer timescales the North Atlantic Oscillation (NAO). For instance, ref.^39^ implicate AMV and NAO in driving local SST and CLLJ changes that led to Mesoamerican drying in the last millennium. Over the instrumental period, the difference between wet and dry years in YP is linked to a tripole SST pattern characteristic of NAO variations (Fig. 2), highlighting the role of North Atlantic SST variability in determining regional precipitation.

## Results

In 2016 we retrieved the inactive stalagmite SN from the Río Secreto (RS) cave system, located in the northeastern YP (20° 35.244′ N; 87° 8.042′ W) (Fig. 1). RS is characterized by extensive semi-inundated underground passages that closely track mean air surface temperature and with relative humidity year-round close to or at 100%. Drip water δ^18^O variability has been characterized from over 10 drip sites in RS and found to closely track rainfall δ^18^O variability, with rainfall integration times from few months to about a year^28,29^ (more details in Methods).

**Figure 3:**
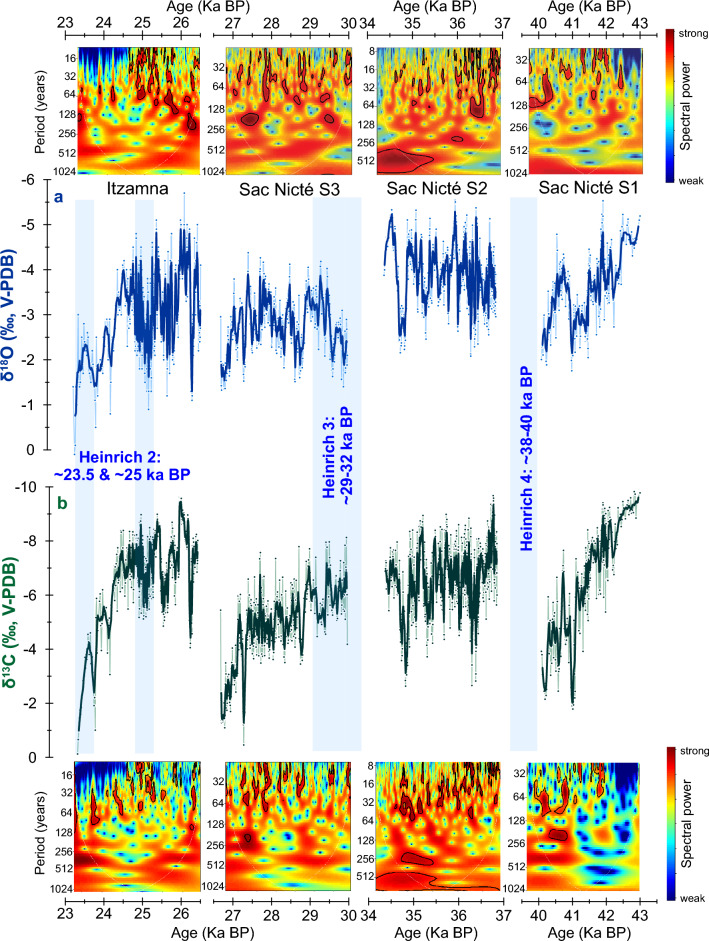
Sac Nicté (SN, this study) and Itzamna^11^ stalagmites oxygen (**A**) and carbon (**B**) isotope records. SN isotopic records cover the interval from 26.6 to 43 ka BP in three growth sections: 40–43 ka BP (section 1), 34.3–36.8 ka BP (section 2), and 26.6–30 ka BP (section 3) ka BP. Sections are separated by two hiatuses. Itzamna isotopic records span 26.5–23.2 ka BP. Timing of Heinrich events 2, 3 and 4 are notated on the plot (blue bars and text). Wavelet spectral power analyses of oxygen and carbon isotopic records are shown at the top and bottom of the plot, respectively. The area outside the white lines indicates the cone of influence where the edge effects of the wavelet transform and uncertainties become important. Black contours reflect statistically significant power spectra (CI = 95%). Wavelet spectral analyses were performed using the R package biwavelet (see Methods and Supplementary for details). Dating uncertainties are less than 1% and therefore have minimal impact on decadal and multidecadal variability interpretations (see Methods).

Following U-Th dating methods^40^, 16 absolute U-Th dates were obtained (Table S3). The chronology was developed based on these 16 dates and 2000 Monte Carlo simulations using the COPRA program (v. 1.16)^41^ (see Methods; Fig. S1) in MATLAB (v. R2021b). The SN isotopic records cover the following time intervals: (1) 40–43 ka BP (section 1), (2) 34.3–36.8 ka BP (section 2), and (3) 26.6–30 ka BP (section 3), with two growth hiatuses between 40 and 36.8 ka BP and 34.3 and 30 ka BP. The first hiatus coincides with Heinrich event 4 (H4), and the last stalagmite section resumed growth after the culmination of H3 (Fig. 3).

The SN stalagmite isotopic records (n = 1416 isotopic analyses, ranging from 0.5 to 2 mm sampling interval) have a temporal resolution of 11 ± 7 years (1 SD) during section 1, of 3.4 ± 1.7 years (1 SD) during section 2, and of 7.9 ± 2 years (1 SD) during section 3 (Fig. S2). Calcite δ^18^O and δ^13^C analyses are reported relative to V-PDB and reproducibility of the standard materials is better than 0.1‰ (more details in Methods and Supplementary). The stalagmite δ^18^O ranges from − 1.7 to − 5.5‰ in section 1, − 2 to − 5.5‰ in section 2, and − 1.3 to − 4.3‰ in section 3. This range is similar to the δ^18^O range of a previously published last glacial stalagmite from the same cave system, known as Itzamna, that spans the interval between 26.5 and 23.2 ka BP (δ^18^O range of − 0.1 to − 5.7‰)^11^.

SN stalagmite δ^18^O values from 43 to 40 ka BP show a positive trend with a statistically significant slope of − 0.7‰ kyr^−1^ (r = 0.61, *p* < 0.01). This positive δ^18^O trend is also associated with a progressive decrease in stalagmite growth rates between 41.5 and 40 ka BP (Fig. S2). Similar statistically significant positive δ^18^O trends are not observed in sections 2 and 3, although the onsets of hiatuses were preceded by progressive growth rate declines and positive δ^18^O and δ^13^C values (i.e., dry conditions, see interpretation below) (Figs. 3 and S2). Finally, we note that the δ^13^C and δ^18^O records closely resemble and are statistically correlated with each other (Fig. S3). All three δ^13^C records show progressive positive trends, especially during sections 1 and 3, with statistically significant slopes of −2.4‰ kyr^−1^ (r = 0.8, *p* < 0.01) and -1‰ kyr^−1^ (r = 0.68, *p* < 0.01), respectively. Stalagmite δ^13^C values range from − 1.7 to − 9.8‰ in section 1, − 2.6 to − 9.6‰ in section 2, and − 0.4 to − 8.1‰ in section 3. The Itzamna stalagmite δ^13^C record from the same cave shows a similar range from − 0.1 to − 9.6‰^11^.

The SN δ^18^O record shows significant variability on decadal and multidecadal timescales. For the first part of section 2 (36.8 and 36 ka BP) where resolution is the highest (i.e., 2.2 ± 1.2 years), wavelet spectral power analysis indicates significant variability between periods of 8 and 128 years (Figs. 3 and S16). The lower resolution sections 1 and 3 also have significant variability between periods of 16 and 128 years (Figs. 3,S14, and S18). Chronological uncertainties are shown to have little effect on these results (see Methods and Supplementary for more details). Spectral analysis of the δ^13^C record sections reveal results like those of δ^18^O record sections (Figs. 3, S15, S17, and S19). Figure 3 illustrates spectral results between the SN and Itzamna δ^18^O records indicating common variability centered between periods of 8 and 128 years^11^.

## Discussion

### Interpretation of proxies

In many tropical regions, including the YP, a negative relationship between rainfall amount and its oxygen isotope composition (i.e., an *amount effect)* has been observed from seasonal to interannual timescales^11,20,21,28–30^. At the RS location, stalagmite δ^18^O is expected to integrate rainfall δ^18^O variability on timescales from few months to about a year, as suggested by multiyear cave monitoring work of rainfall and multiple drip sites^28,29^ (see Methods for more details). Evaporation does not significantly affect the rainfall δ^18^O signal as it is transferred to drip sites, and cave temperature is relatively constant over the course of a year (± 0.3 °C), being 1–2 °C colder than mean annual surface temperature (~ 26 °C)^29^. To verify precipitation at or near isotopic equilibrium conditions with drip water, we performed a Hendy Test^42^ on ten growth profiles of SN (Figs. S12, S13, Table S4). SN δ^18^O values remained relatively constant along each growth layer (Fig. S13, Table S4), passing the Hendy Test. Thus, taking into account: (1) the characteristics and physicochemical conditions of the RS cave system^29^; (2) the Hendy Test^42^ (Figs. S12, S13); (3) pseudo-replication with other stalagmite stable isotope records (Fig. 3); and (4) isotopic equilibrium calculations of more recent specimens from this same cave^43^, we suggest that the SN stalagmite was precipitated under or near isotopic equilibrium conditions with drip water, and thus records rainfall amount variability, as has been proposed by previous studies from the same cave e.g.,^11,30^.

In the RS cave system, precipitation is likely the primary driver of δ^13^C variability, as suggested by previous stalagmite trace element and stable isotope records spanning the mid-Holocene from this cave^19^. While vegetation can also impact speleothem δ^13^C via the relative proportion of C4 versus C3 plants^19^, vegetation type above low latitude caves is expected to be relatively stable over time in the absence of extensive anthropogenic disturbance^18^. Thus, SN δ^13^C variability tracking δ^18^O likely reflects precipitation variability via its control on soil moisture and productivity, CO_2_ degassing, PCP, and bedrock contributions, all which lead to increased stalagmite δ^13^C during times of decreased precipitation^18,19^.

### Decadal to multidecadal variability

The SN stalagmite precipitation variability from decadal (8 years) to multidecadal timescales (64 years) likely reflects a potential connection with the dominant pattern of North Atlantic SST variability associated with AMV. Available paleoclimate, modeling, and instrumental data suggest a connection between hydroclimate variability in the Atlantic Basin and the AMV^39,44,45^. Regarding the Gulf of Mexico and Caribbean regions in particular, a positive correlation between AMV and rainfall has been observed^39^. AMV likely influenced hydroclimate responses in the Atlantic Basin by enhancing a SST gradient across the equator^46^ and modulating the convective activity associated with the ITCZ^45^, in addition to influencing the intensity and rainfall contributions of North Atlantic tropical cyclones^31^. The consistency of the dominant variability periods observed in Holocene and last glacial hydroclimate records from the YP (i.e., between 8 and 64 years) supports an important role of AMV regardless of the climate state (interglacial versus glacial)^11^.

### Millennial variability

#### Correlation with greenland

**Figure 4:**
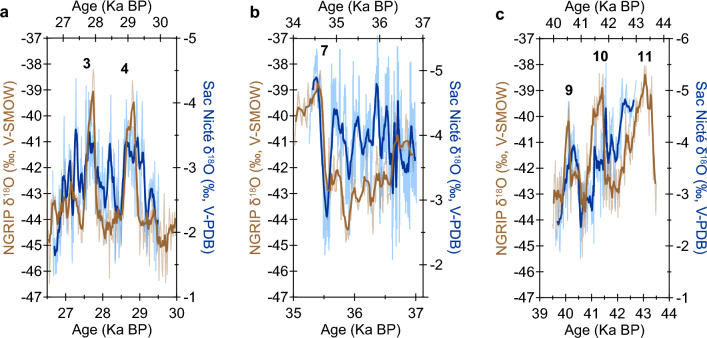
Sac Nicté (SN) δ^18^O record (blue) and the NGRIP ice core δ^18^O record^2^ (brown) over section 3 (**A**), section 2 (**B**), and section 1 (**C**). Black numbers represent NGRIP DO events 3–11. Lighter blue and brown lines represent the SN and NGRIP δ^18^O data (respectively), and the darker lines in front are the running averages with matched resolution of ~100 years. SN chronology was offset within the reported NGRIP age uncertainties of 4–7%^50^ (see Table S1 for chronological shift calculations). For reference, taking the lower-end age uncertainty at 4%, estimated error within the NGRIP chronology is ± 1400 at 35 ka BP and ± 1600 at 40 ka BP. The lower X-axis represents the NGRIP δ^18^O record and the upper X-axis represents the SN δ^18^O record.

Spanning DO events 3–4, 7, and 9–11, and covering Heinrich events 3–4, the SN specimen provides the first YP stalagmite precipitation record defining decadal to millennial timescales during the last glacial period. The SN δ^18^O record resembles the general pattern of the NGRIP δ^18^O record^2^ when their reported chronological uncertainties are taken into account (Fig. 4; Table S1), and spectral coherence plots show statistically significant high wavelet correlations (Fig. S22). This resemblance suggests millennial-scale climate teleconnections between the North Atlantic high latitudes and the Caribbean region, supporting previous studies^5,13,14,22–24,47^. Between 36.8 and 36 ka BP (speleothem section 2), the SN δ^18^O record has the highest resolution (i.e., 2.2 ± 1.2 years) and decadal-to-multidecadal variability clearly dominates the record; thus, visual correlation between the SN and NGRIP records is weak during this time (Fig. 4). It is important to note, however, that these records (1) represent different climate parameters (precipitation versus temperatures), (2) have unique underlying drivers, (3) come from distal geographical and climatological regions, (4) represent different temporal resolutions, (5) have distinct time integrations, and (6) contain individual proxy and chronological uncertainties^2^. Furthermore, a recent study reports significant differences even between coeval Greenland ice core records^48^.

#### Sawtooth pattern

**Figure 5:**
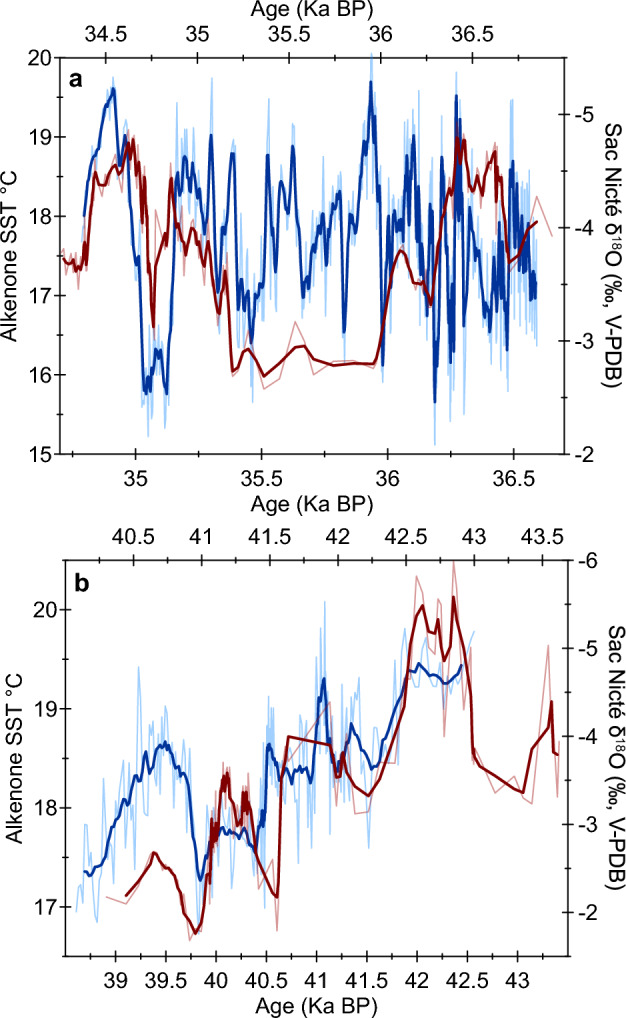
Sac Nicté (SN) δ^18^O record (blue) and alkenone sea surface temperature record from the Bermuda Rise^49^ (red) over section 2 (**A**) and section 1 (**B**). The SST record does not extend beyond 30 ka BP. Lighter blue and red lines represent the SN δ^18^O and SST records (respectively), and the darker lines in front are the running averages with matched resolution of ~30 (section 2) and ~90 (section 1) years. The chronology of SN was offset well within the age uncertainties of the SST record (see Table S2 for chronological shift calculations). The chronological uncertainty of the SST record is associated with that of the Greenland Ice Sheet Project 2 ice core δ^18^O record of 5%^49^. The lower X-axis represents the SST record, and the top X-axis represents the SN δ^18^O record. Lack of correlation between these records is only observed in the interval between ~ 35.2 and 36 ka BP. These records can be reconciled over this time when the ~5 times higher temporal resolution of the SN record relative to the Bermuda Rise SST record is taken into account (see Fig. S7 for details).

The SN precipitation record does not show the characteristic DO sawtooth pattern. Instead, it suggests a slow increase in tropical precipitation during abrupt Greenland warming and a more abrupt precipitation decline coinciding with Greenland progressive cooling (Fig. S4). The nearby lacustrine sedimentary magnetic susceptibility record from Guatemala^23^, and stalagmite proxy records from Cuba and Puerto Rico^13,14^ also resemble the pattern suggested by the SN record (Figs. S4–S6). YP and northern Caribbean patterns of precipitation variability likely reflect North Atlantic SST variability, the underlying hydroclimate driver on millennial timescales. This is supported by visual correlation and spectral coherence plots between the SN δ^18^O record and the Core MD95-2036 alkenone SST record from the Bermuda Rise^49^ (Figs. 5, S7, and S23; Table S2). Similar to the pattern of precipitation variability, last glacial North Atlantic mid-latitude ocean temperature changes are more gradual relative to DO cycles that occur within few years. The sharp abruptness seen in Greenland temperature oscillations has been modelled to occur as a regional phenomenon due to changes in atmospheric circulation and sea-ice coverage^50^, and it is therefore not replicated by tropical SSTs and hydroclimate.

#### Tropical hydroclimate driven by shifts in the position of the ITCZ

NH tropical precipitation reduction during stadial and Heinrich events have been generally linked to a southward shift in the mean position of the ITCZ and its belt of convective activity^4,5,22–24^. Although precipitation in the YP today is enhanced during the summer when the ITCZ lies in its northernmost position, the YP (~ 17–21° N) lies above the convective center of the ITCZ (~ 7° N)^32^ and is indirectly influenced by it. During the summer, moisture is transferred to the YP from the Caribbean via the CLLJ, tropical cyclones, and easterly waves^26,27,31^.

While it has been proposed that the ITCZ simultaneously controls precipitation regimes in both northern South America (Cariaco Basin) and the YP^51^, paleoclimate and instrumental records from these regions suggest climate regimes that are not fully analogous^11,43^. Modeling studies, on the other hand, pose physical limits to the magnitude of past shifts in the mean position of the ITCZ and suggest that potential mean ITCZ shifts per se were likely insufficient to explain the inferred interhemispheric pattern of tropical and subtropical hydroclimate variability during DO and Heinrich events^52^. A previous study based on a last glacial stalagmite δ^18^O record (i.e., Itzamna) from the RS cave^11^ suggests a decrease in the intensity of tropical convective activity associated with the ITCZ without a significant shift in the ITCZ position, in support of modeling studies^52^. Regardless, the SN isotopic records support an anti-phase rainfall amount relationship across the equator during Heinrich events. SH speleothem records indicate higher precipitation during H2–H4 when the SN and Itzamna stalagmites from RS show growth interruptions preceded by positive carbon and oxygen isotopic excursions (i.e., decreasing precipitation) (Figs. S8–S10, SS22)^15–17^.

#### Drivers of YP precipitation variability on millennial timescales

Our results support North Atlantic SST variability as the underlying driver of regional precipitation variability on millennial timescales in the YP (Fig. 5). While locations directly influenced by the ITCZ may reflect a mean ITCZ shift^5,15^, we suggest that changes in the intensity of convective activity associated with the ITCZ due to North Atlantic cooling are captured by the SN isotopic records. Weaker convection associated with the ITCZ, mainly due to cooler SSTs, would reduce the amount of moisture available to the CLLJ, tropical cyclones, and easterly waves. Cooler SSTs may also lead to reduced tropical cyclone activity in the Atlantic. Other contributing factors, such as decreased atmospheric moisture content due to North Atlantic atmospheric and ocean cooling^49^ during stadial and Heinrich events likely also played a role in driving rainfall reductions in the northern Caribbean. The opposite hydroclimate pattern likely happened during warm interstadials. Temperature-vegetation feedbacks could have also contributed to the observed pattern of precipitation variability in the YP. Pollen and charcoal records from Lake Petén Itzá, Guatemala, suggest that significant shifts in temperature and relative abundance of pollens occurred in northern Central America during the last glacial, with temperatures 2–4 °C colder relative to the early Holocene^22^. Climate model simulations of the YP suggest, furthermore, that summer precipitation decreases up to 20% can result from forest cover reduction alone^53^.

Decreased precipitation fluxes from tropical cyclones (TCs) during colder stadial states would additionally contribute to regional precipitation decline. Observational data indicates that up to 20% of summer precipitation along the Mexican Gulf Coast is from landfalling TCs^54^. A statistically significant correlation has also been observed between a stalagmite δ^18^O record from the YP and the frequency of TCs during the Late Holocene^43^. Tropical storm activity is expected to be influenced by Atlantic SSTs. Higher SSTs promote more favorable conditions for their development and intensity^55^. As expected, model simulations indicate that the favorability of tropical cyclogenesis during Heinrich 1 was reduced over the main development region in the Atlantic^56^.

#### Negative feedback mechanism

DO events are associated with climate reorganizations driven by shifts in the strength of AMOC as described by the interhemispheric thermal oceanic bipolar seesaw hypothesis^8,9^. Changes in salt or heat inputs into the North Atlantic alter the strength of AMOC, impacting ocean and atmospheric heat advection and dynamics. AMOC collapse or slowdown reduces northward heat advection from the SH, resulting in North Atlantic cooling and a coeval warming of the South Atlantic^9,10^. Mounting palaeoceanographic evidence suggests that increases in AMOC strength and NADW formation coincide with Greenland interstadial conditions, while decreases in AMOC and NADW formation coincide with Greenland stadials^10^. Evidence of rapid hydrological shifts occurring in a matter of decades and resulting from abrupt atmospheric responses to AMOC has been found across the tropics and subtropical regions of Asia, Africa, the Americas, and Indo-Pacific e.g.,^4^.

Previous studies have proposed that reduced North Atlantic low latitude precipitation resulting from AMOC slowdown/shutdown could consequently impact NADW formation via a negative climate feedback that involves changes in North Atlantic Ocean salinity^5,11,57^. That is, during Heinrich events and the ensuing North Atlantic cooling, decreased Caribbean precipitation would raise its salinity and salt transport to the high latitudes, ultimately invigorating AMOC^57^. Significant precipitation reductions in the Caribbean regions during Heinrich events 2–4, suggested by the speleothem records from the YP, could have thus helped counteract AMOC slowdown. Comparison of the SN δ^18^O record with the Core MD95-2036 alkenone SST record from the Bermuda Rise^49^, an ice-rafted debris (IRD) stack representing a record of iceberg and freshwater input to the North Atlantic^7^, and a Pa/Th record of AMOC strength^58^, are consistent with this mechanism (Fig. S11). However, this negative feedback mechanism involving AMOC and tropical hydroclimate is unlikely to help mitigate future low latitude precipitation reduction over the twenty-first century, considering that the modern hydroclimate driver is not expected to be AMOC slowdown but rather an intensification and movement of the subtropical high pressure system as the concentration of atmospheric greenhouse gases continues to increase^38^.

## Conclusions

The last glacial SN stalagmite δ^18^O and δ^13^C records from the YP reveal dominant decadal to multidecadal variability potentially linked to AMV and NAO, similar to other records from the region spanning the Holocene and last glacial. The YP experienced persistent precipitation reduction that was severe enough to terminate drip water supply to the speleothems during Heinrich events 3 and 4. Although drier conditions during these events are also suggested by other Caribbean paleoclimate records, the evidence from the northeastern YP, located at the climate boundary between tropical and subtropical conditions, suggests greater precipitation deficits over the northwestern Caribbean at the time when cooler ocean and atmospheric conditions extended over the North Atlantic region. Last glacial Caribbean hydroclimate variability on millennial timescales does not show the typical sawtooth pattern of DO cycles in Greenland, but a pattern that resembles that of North Atlantic SST variability, with higher precipitation during the warm North Atlantic phase and lower precipitation during the cold phase. Because of the distal location of the YP relative to the ITCZ and its belt of convective activity, precipitation variability in the YP and the northern Caribbean region probably cannot be explained solely as the result of a shift in the mean position of convective activity associated with the ITCZ. Rather, changes in ITCZ position likely had an indirect impact, with the underlying hydroclimate driver on millennial timescales being North Atlantic SST variability impacting convective activity.

Our results provide several implications for studies of future climate in the region. First, we present new insight on long-term hydroclimate dynamics in the northern Caribbean region (particularly during an interval of frequent, abrupt climate change) that will complement model projections of future precipitation change with empirical information. Additionally, our records provide the most alarming examples yet of persistent precipitation decline in the YP and northern Caribbean region from a speleothem, highlighting the modern vulnerability of the region to climate trends. Our results can thus inform studies investigating future change at climate boundaries, for example between tropical and subtropical conditions as in the case of the YP.

Lastly, our results are compatible with the existence of a negative climate feedback between AMOC and low latitude hydroclimate, supported by previous studies^5,57,59,60^. Caribbean precipitation reductions during stadial events and Heinrich episodes when AMOC slowed down or collapsed would have enhanced ocean salinity, in turn invigorating AMOC^5,57^. Recent studies demonstrate that AMOC has slowed down since the mid-twentieth century, with further weakening expected as warming continues^61^. While significant rainfall reduction is projected to occur in the broader Caribbean and Gulf of Mexico regions, including the YP, by the end of the twenty-first century, this is expected to occur due to an altered subtropical high pressure system^38^. Ergo, this proposed negative climate feedback mechanism between AMOC and North Atlantic low latitude hydroclimate is unlikely to help mitigate future tropical precipitation reduction over the twenty-first century.

## Methods

### Dating, isotopic, and spectral analyses

The SN stalagmite carbon and oxygen isotope composition was determined in the Stable Isotope Laboratory at UMass Amherst and in the Paleoclimatology Laboratory at Auburn University using an on-line carbonate preparation system linked to a Thermo Scientific Delta Plus isotope ratio mass spectrometer. Reproducibility of the standard materials is better than ± 0.1‰. All values are reported relative to the V-PDB standard^62^. We built the chronology of SN using the Uranium-series dating technique^40^. Calcite powders, each weighing between 0.1 and 0.3 g, were collected from the speleothem using a micromill. After collection, Uranium and Thorium isotopic values were measured using a multi-collector inductively coupled plasma mass spectrometer (MC-ICP-MS) in the Paleoclimate and Geochronology Laboratory at the Massachusetts Institute of Technology (MIT). Before measuring, samples were prepared for analysis and Uranium and Thorium were separated following dating methods^40^. The age models for sections 1–3 were developed with 2000 Monte Carlo (MC) simulations using the COPRA program^41^ (v. 1.16) in MATLAB (v. R2021b). (Fig. S1). Wavelet spectral analysis, cross-correlation, and wavelet correlation analysis were performed using the R package biwavelet (Figs. 3, S3, and S14–S19)^63–65^. Unevenly spaced time series of δ^18^O and δ^13^C were interpolated by the Akima splines method^66^. Akima is one of the most common, useful, and effective methods to interpolate unevenly spaced paleoclimate time series^67,68^. However, we have corroborated whether this interpolation method works properly with our data comparing the interpolated time series with the original unevenly spaced time series, and the differences are negligible. We have used the R package Pracma^69^ that includes the Akima method to interpolate the δ^18^O and δ^13^C time series. Plots of these results (Figs. 3; S14–S21) are interpreted by looking for clusters spanning the same time interval. Our results indicate that the record has dominant periods as short as 8 years and as long as 128 years, with the periods in between these two extremes possible. Higher intensity of the color is indicating more power over a specific periodicity range within that same time interval. This indicates that periodicity range in particular (e.g., 8–16) is more strongly represented in the timeseries.

### Effect of chronological uncertainty on spectral results

As explained above, the spectral results we present in this study are based on the mean time series derived from 2000 Monte Carlo simulations that take into consideration the U-Th dating uncertainties; therefore, they are based on the stalagmite timeseries with the highest probability within the uncertainty distribution. However, while chronological uncertainties are less than 1% and thus should have little impact on decadal to multidecadal variability interpretations, we assess the potential impact of chronological uncertainties on our spectral results by expanding and contracting the timescale, following previous methods^11^. For the multidecadal periods of variability, e.g., centered at ~32 and ~64 years, we use the entire interval of each section and expand/contract the timescale by the maximum age uncertainty per section: ± 280 years (section 1), ± 400 years (section 2), and ± 140 years (section 3). This results in periods of variability of 32 ± 2 and 64 ± 4 years (section 1), 32 ± 6 and 64 ± 11 years (section 2), and 32 ± 2 and 64 ± 3.5 years (section 3). For the decadal variability in SN at ~8 years, we expand and contract the timescale over an 800-year time interval between 36.0 and 36.8 ka BP of section 2. To do this we apply the average age uncertainty (± 205 years) and determine the impact on the dominant period of variability. This results in dominant periods of 10 and 6 years on the expanded and contracted timescales, respectively (i.e., ~8 ± 2 years). The results from this approach are thus consistent with our previous results based on the mean time series derived from Monte Carlo simulations. To further verify the SN decadal variability as short as ~8 years in section 2, we performed an additional test of expanding/contracting the timescale by the average age uncertainty for the section (± 205 years); however, we did this only for certain ages during the beginning of section 2 where higher resolution allows the consistent observation of decadal variability (i.e., ~36.2 and ~36.7 ka BP; see Fig. S16). We found that variability of ~8 ±  < 1 years continues to appear during the older part of section 2 despite the expansion/contraction, in agreement with our decadal interpretation. All three panels show significant decadal and multidecadal variability at times around and after 36.2 ka BP (Fig. S21).

To increase confidence in our decadal to multidecadal interpretations, we also performed wavelet spectral analysis on the two stalagmite δ^18^O timeseries that represent the boundaries of the 95% confidence interval around the mean derived from 2000 Monte Carlo simulations, as described in the methods above. We selected these two timeseries, one representing the oldest timeseries (hi boundary) and the other the youngest (low boundary). We note that because they represent the boundaries of the confidence interval, they thus represent the least likely (probabilistically) curves to represent the data. We obtained approximately equivalent wavelet results for all three timeseries (mean, high and low) (Fig. S20). These analyses suggest that in the case of the SN speleothem records, chronological uncertainties have a minimal influence on the dominant periods of variability obtained from spectral analysis.

Lastly, we propose that U-Th uncertainties are not necessarily directly transferable to stalagmite growth rate variability. That is, stalagmite growth rates are not expected to vary as significantly as some age uncertainties would imply if taken at face value. We argue that stalagmite growth rates from the study site do not vary significantly over time because the underlying processing controlling calcification are not expected to change significantly. In areas and regions with extremely thin soils and therefore little soil carbon content, such as in the study area and large portions of the YP, stalagmite calcite precipitation is strongly controlled by water/rock interactions (as reflected by evidence from positive stalagmite δ^13^C values) and thus not expected to change significantly over time (because it is relatively insensitive to potentially highly variable biological processes at the surface). The other aspect that could exert a control on stalagmite calcification rates in our site is shifts in CO_2_ degassing rates over time, which reflect alkalinity and total dissolved inorganic carbon (DIC), as well as the CO_2_ partial pressure gradient between dripwater and cave air^70^. We would not expect this gradient to change much over time in our study site, however. The study cave chamber is poorly ventilated (far from cave openings to the surface) and pristine (without the influence of significant human, animal, plant, and microorganismal life). Therefore, the total amount of dripwater DIC (controlled mostly by rock/water interactions) and CO_2_ degassing rates are not expected to vary significantly over time at our study site. Compatible with this hypothesis, Sac Nicté stalagmite growth rates range from 0.1 to 0.3 mm/yr (3 × difference) in section 3, 0.2–2 mm/yr in section 2 (10x), and 0.1–1 mm/yr (10x) in section 1. These growth rate results are not unexpected, and relatively constant growth rates are also observed in other speleothems from the YP region, such as we reported for example in ref.^43^ via laminae counting of the Chaac specimen (~1 mm/year) and in ref.^19^ where we presented 500-year stalagmite trace element and isotopic records that show visually distinctive annual laminations (~0.5–2 mm/year).

### Amount effect and cave monitoring

The Río Secreto cave system has been monitored continuously since 2014 to characterize air relative humidity, air and groundwater temperature, and the δ^18^O composition of drip water^11,28–30^. Monitoring evidence indicates that drip water at various drip sites within the cave system has some of the shortest rainfall integration times observed in caves worldwide, of only 3–18 months. Evaporation was found to not significantly affect the rainfall isotopic signal as it is transferred to drip sites from the surface^28,29^. Relative humidity in the Río Secreto chamber where SN was collected was consistently at or near 100%, and average air temperature over the course of a year was 24.6 ± 0.3 °C, thus significantly modulating mean annual air temperature variability outside the cave chamber (25.9 ± 14 °C)^29^. Evaporation at the surface and inside the cave system has little to no effect in altering the isotopic composition of rainfall by drip water due to (1) drip water isotopic values similar to the Local Meteoric Water Line; (2) drip water isotopic composition similar to coeval rainfall; (3) drip water annual average δ^18^O composition similar the annual amount-weighted δ^18^O composition of rainfall^28,29^. In tropical regions, and in the YP, the existence of the amount effect relationship between rainfall amount and its δ^18^O composition has been observed^11,20,21,28–30^. While we have no means to determine the existence and magnitude of an amount effect on seasonal or interannual timescales during the last glacial for the YP, we can assess the extent to which the amount effect varies over vast regions today to get a sense of its geographical variability (i.e., potentially reflecting the effect of different moisture sources and climate dynamics). In ref.^11^ we show that the slope of the amount effect relationship that describes the change in rainfall δ^18^O (‰) per mm of rainfall amount change does not vary significantly over vast regions including the Gulf of Mexico and Caribbean, based on instrumental rainfall isotopic data^11^. Furthermore, in the YP, annual and multidecadal rainfall δ^18^O variability reflects mostly a single dominant tropical Atlantic/Caribbean source^21^. In support of this, the SN stalagmite δ^13^C and δ^18^O records, reflecting mostly local rock-water interactions and the amount effect, respectively, would not be expected to correlate with each other as observed if the influence of moisture source changes controlled annual and interannual rainfall δ^18^O variability in the past. Lastly, we do not discard the possibility that last glacial boundary conditions could have produced a mean rainfall δ^18^O shift across the North Atlantic relative to mean rainfall δ^18^O values today. During the last glacial time interval, large continental ice volume was associated with an ocean δ^18^O composition 1.2‰ higher than today, and North Atlantic rainfall δ^18^O may have been 1.6‰ higher compared to modern values, as global circulation model experiments suggest^71,72^.

### Supplementary Information

Supplementary Information.

## Data Availability

Supplementary information accompanies this paper at http://www.nature.com/scientificreports. All datasets will be submitted to the NOAA Paleoclimatology database (https://www.ncdc.noaa.gov/data-access/paleoclimatology-data/datasets) upon publication but are available from corresponding author upon reasonable request.
