# Supplementary material for: Last glacial hydroclimate variability in the Yucatán Peninsula not just driven by ITCZ shifts
Source: Sci Rep. 2023 Sep 1;13:14356. doi: 10.1038/s41598-023-40108-6 (PMC10474098; doi:10.1038/s41598-023-40108-6)
Supplement: Supplementary file 1 — Supplementary Information. [file 41598_2023_40108_MOESM1_ESM.pdf]

Last glacial hydroclimate variability in the Yucatán Peninsula not just driven by ITCZ shifts

Leah Travis-Taylor, Martín Medina-Elizalde, Ambarish V. Karmalkar, Josué Polanco-Martinez, Gabriela Serrato Marks, Stephen Burns, Fernanda Lases-Hernández, and David McGee

Supplementary Information

Tables:

Values of chronological shifts for Figure 3: Sac Nicté plotted with NGRIP

The chronology of Sac Nicté was offset for best correlation with the NGRIP record. Correlated chronology is within the age uncertainties reported for the NGRIP ice core record of 4-7%[1]. For reference, taking the lower-end age uncertainty at 4%, estimated error at certain dates within the NGRIP chronology is ±1400 at 35 ka BP and ±1600 at 40 ka BP. Error in the SN chronology is approximately ≤1%.

Section #	Value of Shift	SN Year	Sac Nicté shifted older/younger
Section 1	~300 years	43 ka BP	Older
Section 1	~400 years	42 ka BP	Older
Section 1	~500 years	40 ka BP	Older
Section 2	~200 years	37 ka BP	Younger
Section 2	~400 years	36 ka BP	Younger
Section 2	~900 years	34.5 ka BP	Younger
Section 3	~300 years	29 ka BP	Older
Section 3	~100 years	28 ka BP	Older
Section 3	~50 years	27 ka BP	Older

Table S1. Values of chronological shifts for comparison plots of Sac Nicté and NGRIP[2].

Values of chronological shifts for Figure 5: Sac Nicté plotted with SST

The chronology of Sac Nicté was offset for best correlation with the SST record. Correlated chronology is within the age uncertainties reported for the SST record. Time scale uncertainty for the SST record is associated with that of the Greenland Ice Sheet Project 2 (GISP2) ice core, which is 5%[3]. For reference, the estimated error in the SST chronology is ±2000 at 40 ka BP and ±1750 at 35 ka BP with 5% uncertainty. Error in the SN chronology is approximately ≤1%.

Section #	Value of Shift	SN Year	Sac Nicté shifted older/younger
Section 1	~400 years	43 ka BP	Older
Section 1	~800 years	42 ka BP	Older
Section 1	~1500 years	40 ka BP	Older
Section 2	~300 years	37 ka BP	Older
Section 2	~50 years	36 ka BP	Older
Section 2	~400 years	34.5 ka BP	Younger

Table S2. Values of chronological shifts for comparison plots of Sac Nicté and SST[3]. The SST record does not extend after 30 ka BP; therefore, section 3 is not reported here as it could not be plotted.

Depth (mm)	238U (ng/g)	± (2σ)	232Th (pg/g)	± (2σ)	234U (per mille)	± (2σ)	(230Th/238U) (activity)	± (2σ)	230Th/232Th (ppm atomic)	± (2σ)
58.7	246.000	5.000	94.000	1.900	17.500	0.700	0.225	0.001	9350.000	40.000
260.7	357.000	7.000	58.900	1.200	22.100	0.700	0.234	0.001	22540.000	80.000
362.4	293.000	6.000	41.300	0.800	28.000	2.000	0.238	0.001	26890.000	100.000
482.0	423.124	8.464	80.178	1.612	24.083	0.829	0.242	0.001	20307.552	66.453
616.0	484.318	9.688	71.756	1.442	29.610	0.945	0.248	0.001	26576.588	84.332
682.0	238.000	5.000	86.000	5.000	40.000	4.000	0.282	0.001	12400.000	700.000
914.0	352.409	7.049	59.701	1.627	33.049	1.267	0.293	0.001	27456.751	523.768
1046.0	388.597	7.773	65.193	1.781	30.764	1.339	0.294	0.001	27838.331	520.614
1201.7	396.735	7.966	179.860	3.625	27.548	5.570	0.293	0.002	10275.048	42.122
1510.0	435.337	8.707	118.238	2.665	31.407	1.343	0.296	0.001	17318.229	181.329
1515.0	182.708	3.654	93.366	2.198	35.057	2.165	0.316	0.001	9818.993	127.106
1653.0	491.254	9.827	26.854	1.110	35.867	1.988	0.329	0.002	95584.107	3493.666
1721.0	394.323	7.888	81.962	1.648	37.801	0.937	0.330	0.001	25170.102	72.238
1785.0	427.213	8.545	68.404	1.379	38.097	0.933	0.332	0.001	32913.726	133.419
1886.0	378.204	7.564	80.191	1.615	35.325	0.779	0.332	0.001	24882.164	83.720
1956.0	383.227	7.668	87.117	1.888	32.934	2.224	0.335	0.001	23398.689	201.021

Depth (mm)	Age (yr) (uncorr)	± (2σ)	Age (yr) (corr)	± (2σ)	$^{234}U_{init}$ (per mil)	± (2σ)	*Final Age (yr B.P.)*	*± (2σ)*
58.7	27210	130	27200	130	18.800	0.800	***27140***	***130***
260.7	28380	110	28370	110	24.000	0.800	***28310***	***110***
362.4	28720	140	28710	140	30.000	2.000	***28650***	***140***
482.0	29420	110	29410	110	26.167	0.901	***29340***	***110***
616.0	30010	110	30000	110	32.226	1.029	***29940***	***110***
682.0	34400	200	34400	200	44.000	5.000	***34400***	***200***
914.0	36300	210	36290	210	36.613	1.404	***36230***	***210***
1046.0	36570	120	36570	120	34.108	1.485	***36500***	***120***
1201.7	36600	400	36590	400	30.545	6.176	***36520***	***400***
1510.0	36854	98	36846	98	34.849	1.490	***36780***	***98***
1515.0	39640	210	39620	210	39.205	2.421	***39560***	***210***
1653.0	41590	280	41580	280	40.333	2.236	***41520***	***280***
1721.0	41550	150	41540	150	42.503	1.054	***41480***	***150***
1785.0	41900	190	41900	190	42.879	1.050	***41830***	***190***
1886.0	42110	160	42100	160	39.781	0.877	***42030***	***160***
1956.0	42650	180	42650	180	37.146	2.509	***42720***	***180***

Table S3. Raw Sac Nicté U-Th data. Final ages used in the age model (Fig. S1) are italicized and shaded in green. Reported errors for ^{238}U and ^{232}Th concentrations are estimated to be ±1% due to uncertainties in spike concentration; analytical uncertainties are smaller. "Uncorrected" indicates that no correction has been made for initial ^{230}Th. Ages are corrected for detrital ^{230}Th assuming an initial ^{230}Th/^{232}Th of $(4.4\pm2.2) \times 10^{-6}$. $\delta^{234}U_{initial}$ corrected was calculated based on ^{230}Th age (T), i.e., $\delta^{234}U_{initial} = \delta^{234}U_{measured}\ X\ e^{\lambda234*T}$, and T is corrected age. B.P. stands for "Before Present" where the "Present" is defined as the January 1, 1950 C.E. Decay constants for ^{230}Th and ^{234}U are from ref [4]; decay constant for ^{238}U is 1.55125×10^{-10} yr^{-1} (ref. [5]).

$\delta^{234}U = ([^{234}U/^{238}U]_{activity} - 1) \times 1000$. $[^{230}Th/^{238}U]_{activity} = 1 - e^{-\lambda_{230} T} + (\delta^{234}U_{measured}/1000)[\lambda_{230}/(\lambda_{230} - \lambda_{234})](1 - e^{-(\lambda_{230} - \lambda_{234}) T})$, where T is age.

Layer Depth	Distance from Growth Axis (mm)	^{18}O	^{13}C
82	6	-1.19	-0.97
82	5	-0.93	-0.91
82	4	-1.21	-0.74
82	3	-1.46	-0.86
82	2	-0.43	-1.01
82	1	-1.13	-0.68
82	0	-1.60	-2.15
82	-1	-1.26	-0.72
82	-2	-0.84	-0.69
82	-3	-1.06	-0.80
82	-4	-1.38	-1.51
82	-5	-2.73	-4.68
390	5	-2.21	-4.01
390	4	-1.91	-3.09
390	3	-2.32	-3.99
390	2	-1.83	-3.35
390	1	-2.14	-3.55
390	0	-1.82	-3.59
390	-1	-2.07	-4.28
390	-2	-2.33	-4.28
390	-3	-2.10	-4.06
390	-4	-1.93	-4.19
390	-5	-2.10	-3.74
586	6	-2.28	-4.73
586	5	-2.36	-5.22
586	4	-1.89	-4.95
586	3	-2.28	-5.63
586	2	-2.23	-5.36
586	1	-2.97	-6.28
586	0	-2.47	-5.63
586	-1	-2.32	-4.89
586	-2	-2.60	-5.30
586	-3	-2.52	-4.55
586	-4	-2.70	-4.25
586	-5	-1.58	-3.12
586	-6	-1.76	-3.00
824	5	-2.78	-7.67
824	4	-2.41	-4.30
824	3	-2.22	-5.69
824	2	-2.61	-6.55
824	1	-2.23	-5.18
824	0	-2.41	-7.28
824	-1	-2.91	-7.73

824	-2	-2.73	-6.65
824	-3	-2.94	-6.56
824	-4	-2.60	-4.62
824	-5	-2.32	-4.15
1068	5	-3.89	-5.98
1068	4	-3.54	-4.46
1068	3	-3.99	-6.59
1068	2	-3.77	-5.73
1068	0	-3.22	-6.34
1068	-1	-3.34	-6.06
1068	-2	-3.45	-5.63
1068	-3	-3.34	-4.54
1068	-4	-3.25	-4.51
1068	-5	-2.87	-5.19
1200	4	-2.36	-5.28
1200	3	-2.32	-4.62
1200	2	-2.62	-4.71
1200	1	-2.50	-4.95
1200	0	-2.99	-5.85
1200	-1	-3.33	-6.35
1200	-2	-2.80	-6.92
1200	-3	-3.42	-7.19
1200	-4	-3.75	-8.37
1200	-5	-3.51	-7.47
1416	5	-2.71	-4.35
1416	4	-2.72	-4.39
1416	3	-2.85	-4.63
1416	2	-2.98	-5.64
1416	1	-3.00	-5.42
1416	0	-4.39	-8.95
1416	-1	-2.88	-5.07
1416	-2	-3.12	-5.96
1416	-3	-2.78	-4.61
1416	-4	-2.96	-5.16
1416	-5	-3.17	-4.97
1416	-6	-2.65	-4.01
1602	5	-1.44	-0.95
1602	4	-1.63	-1.20
1602	3	-1.19	-1.66
1602	2	-1.45	-1.64
1602	1	-1.45	-2.21
1602	0	-2.53	-6.19
1602	-1	-1.67	-2.43
1602	-2	-1.76	-2.51

1602	-3	-1.73	-2.23
1602	-4	-1.68	-2.28
1602	-5	-1.87	-2.14
1876	7	-2.60	-6.70
1876	6	-2.17	-4.55
1876	5	-1.95	-4.05
1876	4	-2.10	-4.08
1876	3	-3.71	-5.45
1876	2	-2.86	-6.97
1876	1	-2.97	-7.77
1876	0	-4.12	-9.20
1876	-1	-2.33	-6.34
1876	-2	-2.46	-5.55
1876	-3	-2.01	-3.76
1876	-4	-2.97	-4.17
1876	-5	-3.55	-5.35
1876	-6	-3.89	-5.40
1962	6	-4.64	-7.22
1962	5	-4.22	-7.77
1962	4	-4.94	-8.08
1962	3	-4.96	-8.43
1962	2	-5.08	-9.09
1962	1	-5.10	-9.24
1962	0	-4.81	-9.22
1962	-1	-5.12	-9.20
1962	-2	-5.16	-8.73
1962	-3	-4.70	-8.21
1962	-4	-4.54	-8.06
1962	-5	-5.14	-9.17
1962	-6	-4.47	-7.81

Table S4. Hendy Test isotopic data for the 10 analyzed growth layers (see Figs. S12-S13 below).

Supplementary Figures:

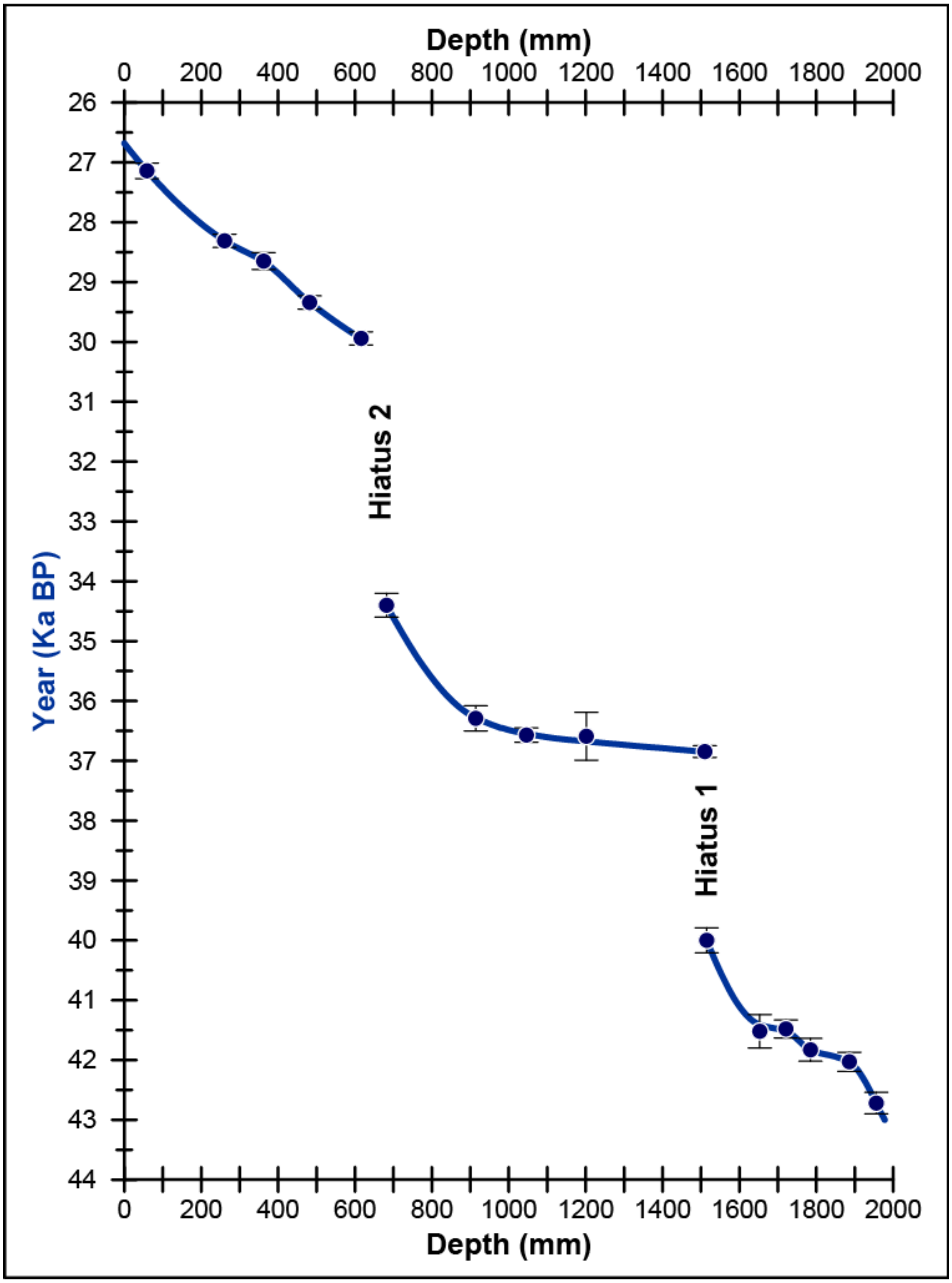

Figure S1. Sac Nicté age versus depth with 16 absolute U/Th dates and associated error bars (<1%). The chronologies for sections 1-3 were created using COPRA in MatLab[6]. Note that no observational hiatus is found between 682.0 - 914.0mm, so this interval in the age model is counted as a time of slow growth rate, rather than a hiatus.

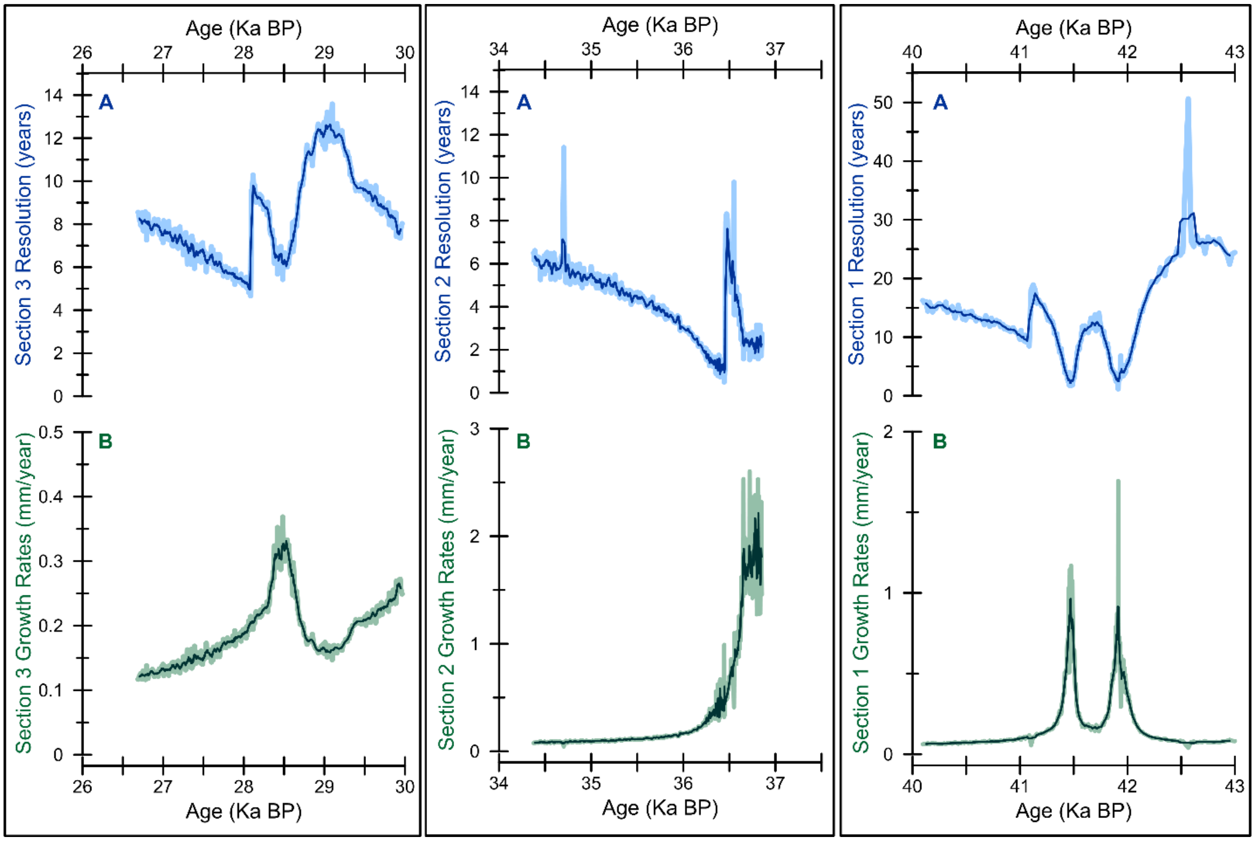

Figure S2. Calculated Sac Nicté (A) resolution and (B) growth rates for sections 1-3.

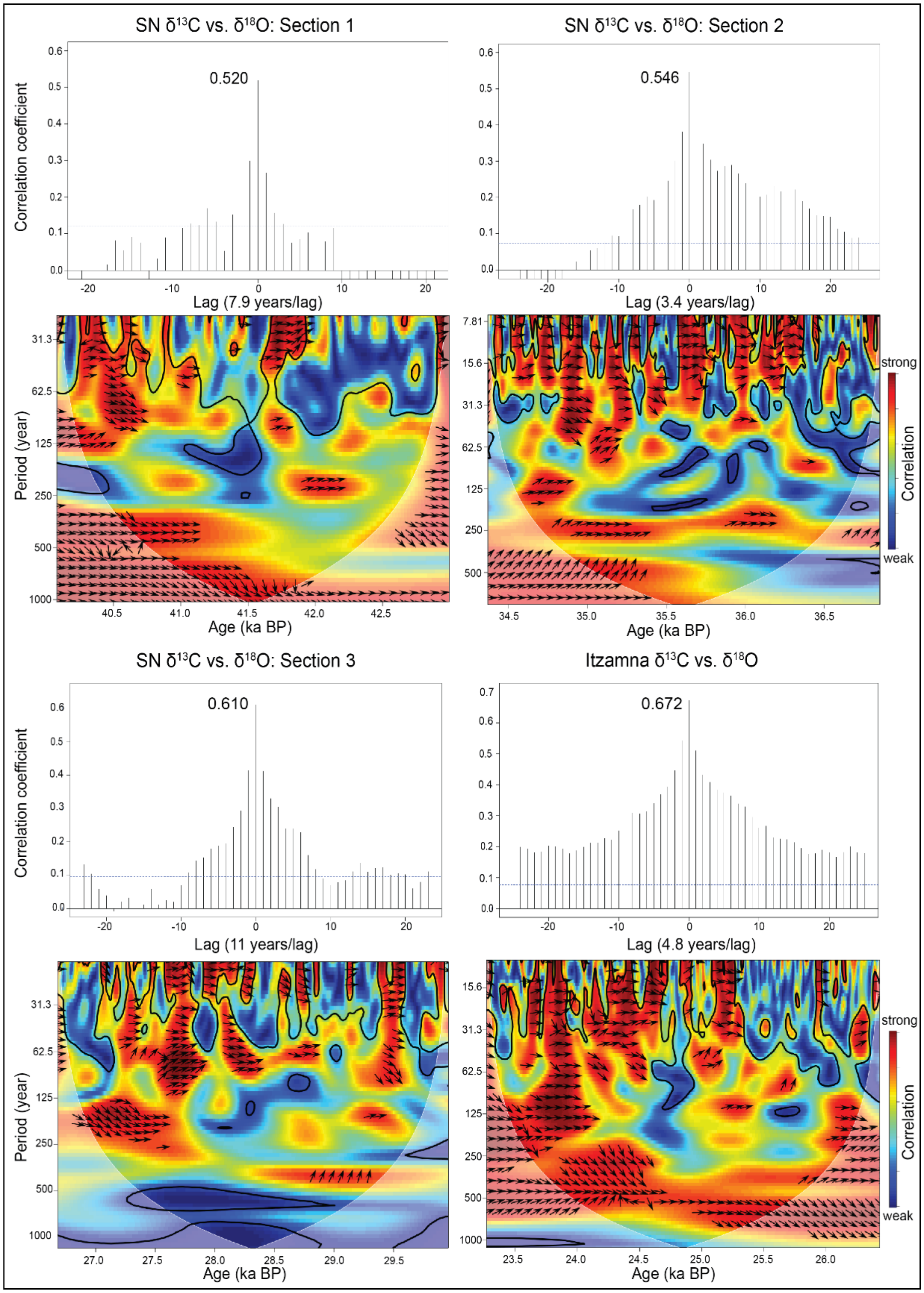

Figure S3. Plot of $\delta^{13}C$ versus $\delta^{18}O$ correlation coefficients for sections 1-3 of Sac Nicté and for Itzamna[7]. The $\delta^{13}C$ and $\delta^{18}O$ of each section are significantly correlated at 0 lag. Correlations were performed using the R package biwavelet[8-10].

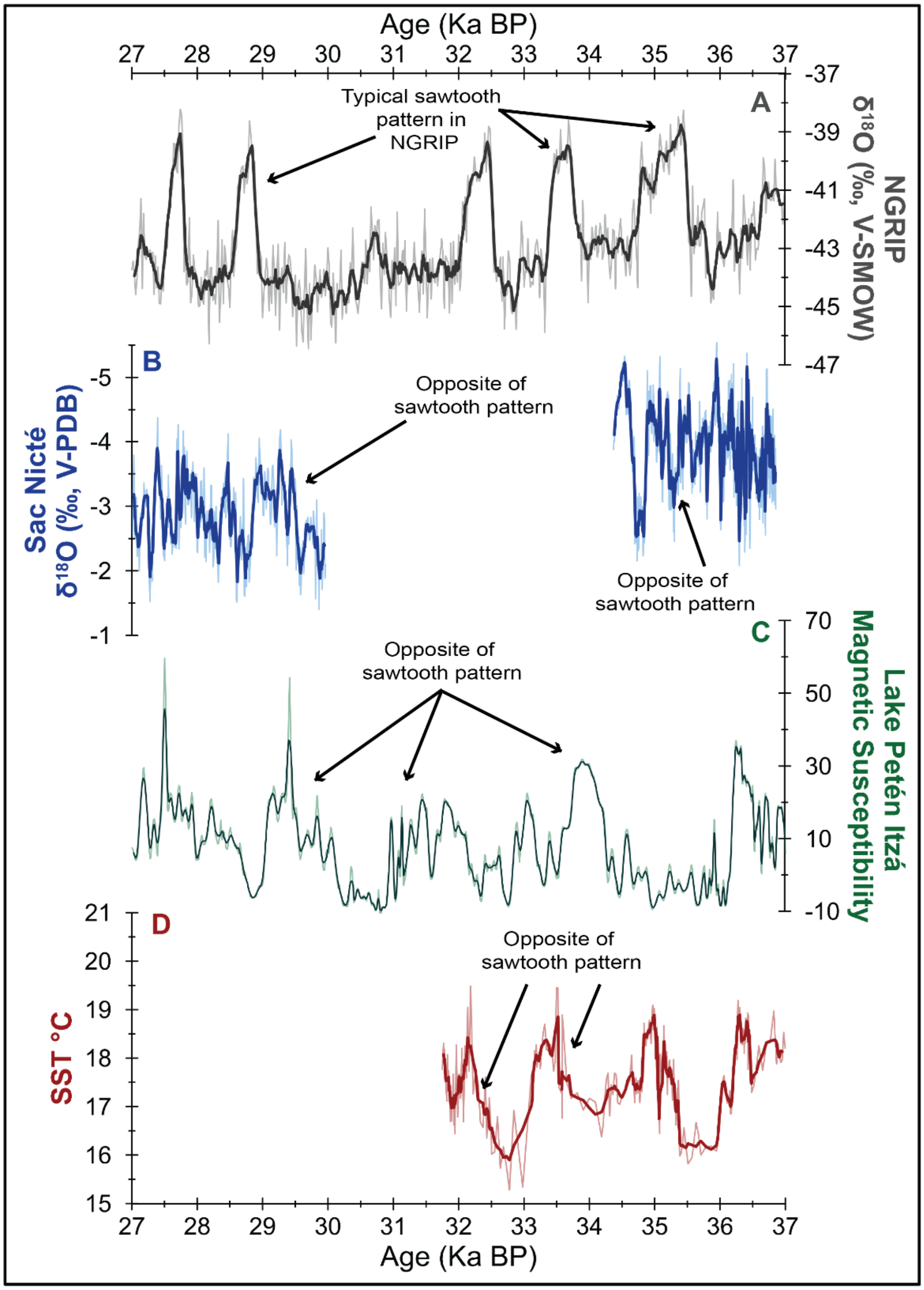

Figure S4. Sac Nicté (A) oxygen isotope record compared with (B) NGRIP[2], (C) Lake Petén Itzá sediment magnetic susceptibility record[11,12], and (D) alkenone temperature record from the Bermuda Rise[3]. We do not see the typical sawtooth pattern of DO cycles in the YP precipitation record. The sharp abruptness seen in Greenland temperature oscillations has been modelled to occur as a regional phenomenon[1] and it is therefore not replicated by tropical SSTs and hydroclimate.

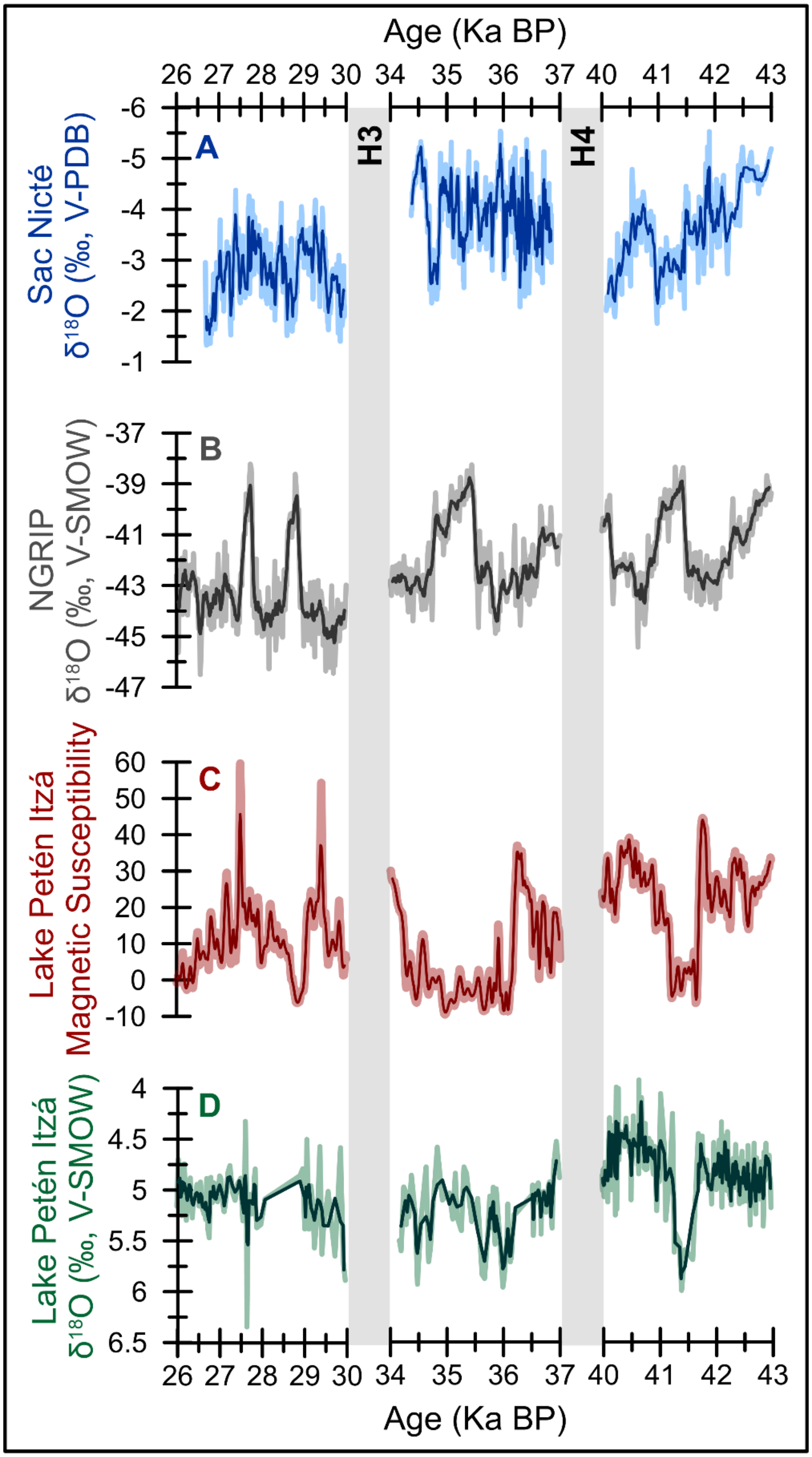

Figure S5. Sac Nicté (A) oxygen isotope record compared with (B) NGRIP[2], (C) Lake Petén Itzá sediment magnetic susceptibility record[11,12], and (D) Lake Petén Itzá ostracod oxygen isotope record[12].

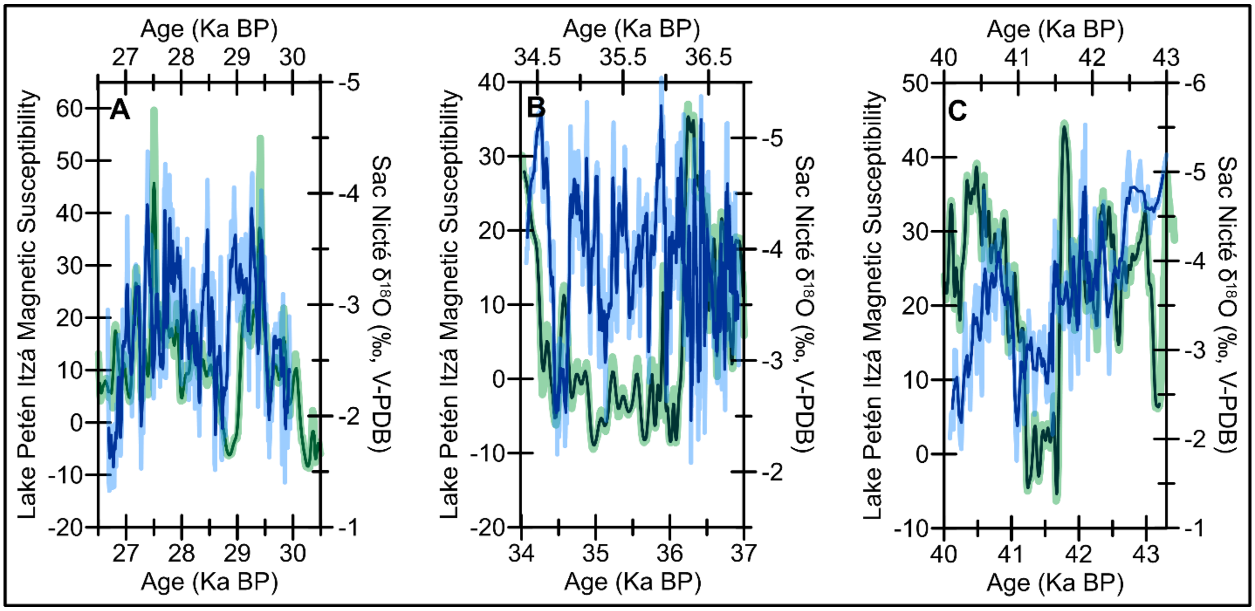

Figure S6. Sac Nicté oxygen isotope record compared with the Lake Petén Itzá sediment magnetic susceptibility record[11,12] over (A) section 3, (B) section 2, and (C) section 1.

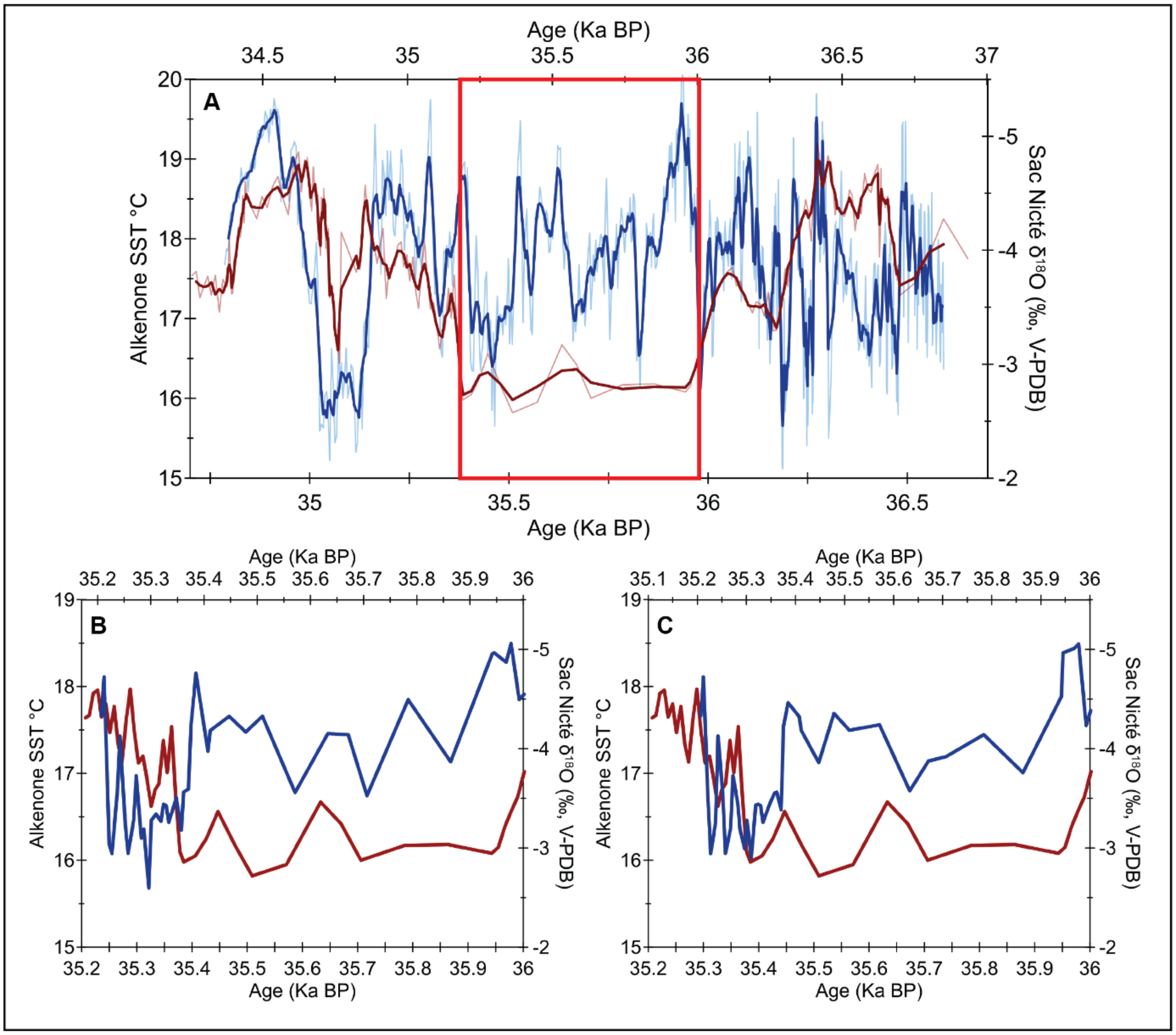

Figure S7. Sac Nicté (SN) record (blue) plotted with the available alkenone temperature record from the Bermuda Rise[3] (red) over section 2 (top – panel A). The light red box designates a window of inconsistency between the two records during this interval. Panels B and C show the SST and SN records incrementally resolution-matched to demonstrate the that inconsistency is due to very low resolution of the SST record during the highlighted interval. Panel B shows incremental resolution matching based on selecting individual values from the SN record only when there is an associated SST value at that date as well, giving the two records nearly identical resolution. In panel C, integration of the all values in between selected SN points where there is an associated SST value is conducted. In all three panels, the chronology of SN was offset for best visual correlation with the SST record chronology within uncertainty (see Fig. 5 and Table S2 for details on panel A). The lower x-axes reflect the SST record chronology, while the upper x-axes reflect the SN record chronology.

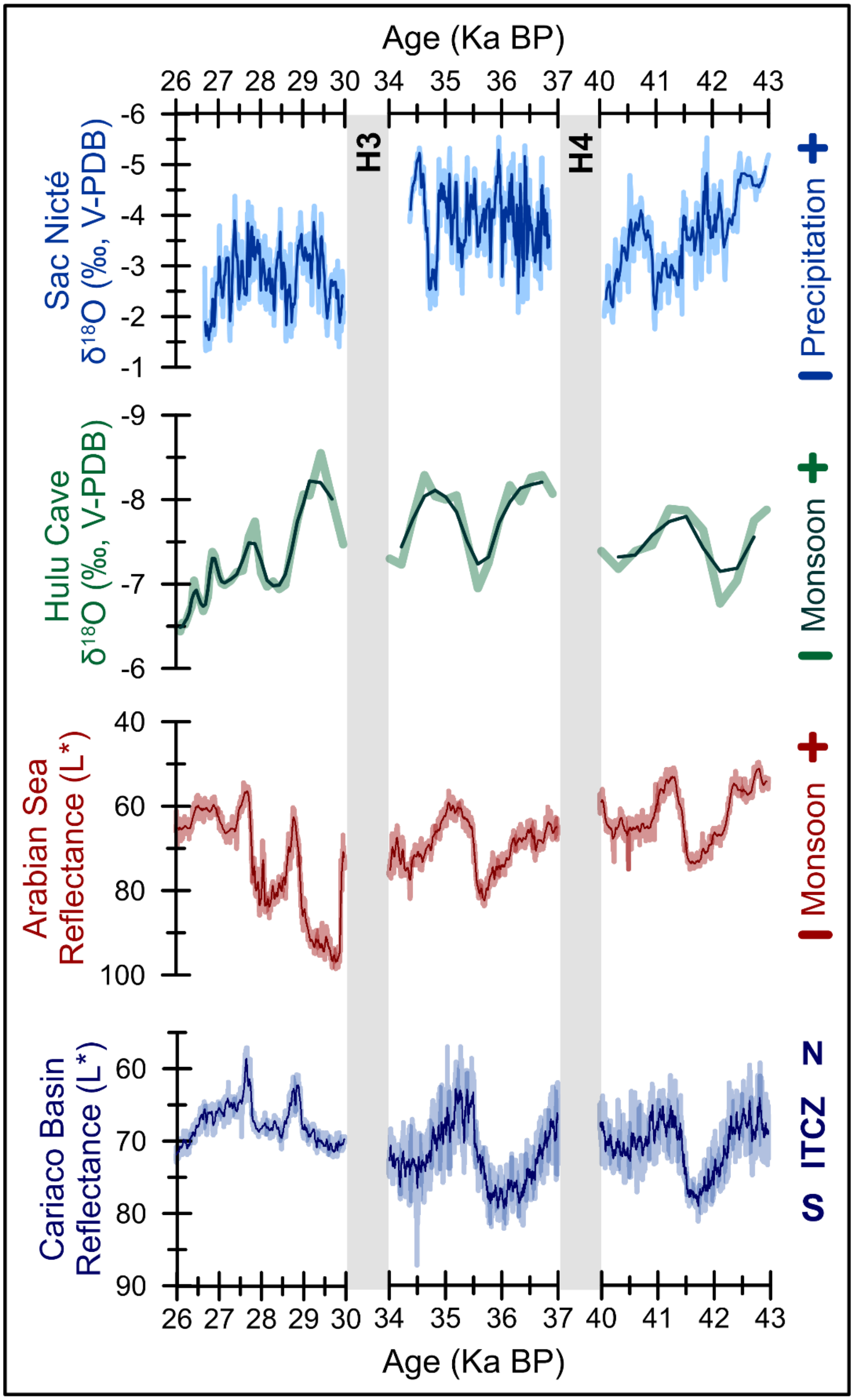

Figure S8. (A) Sac Nicté record compared with records from the Northern Hemisphere over the same time interval – (B) Hulu Cave oxygen isotope record[13], (C) Arabian Sea reflectance record[14], and (D) Cariaco Basin reflectance record[14]. Inconsistencies in the timing of Heinrich events among these records are likely due to chronological uncertainties of 0.7-1.5%[13] and >200 years[14].

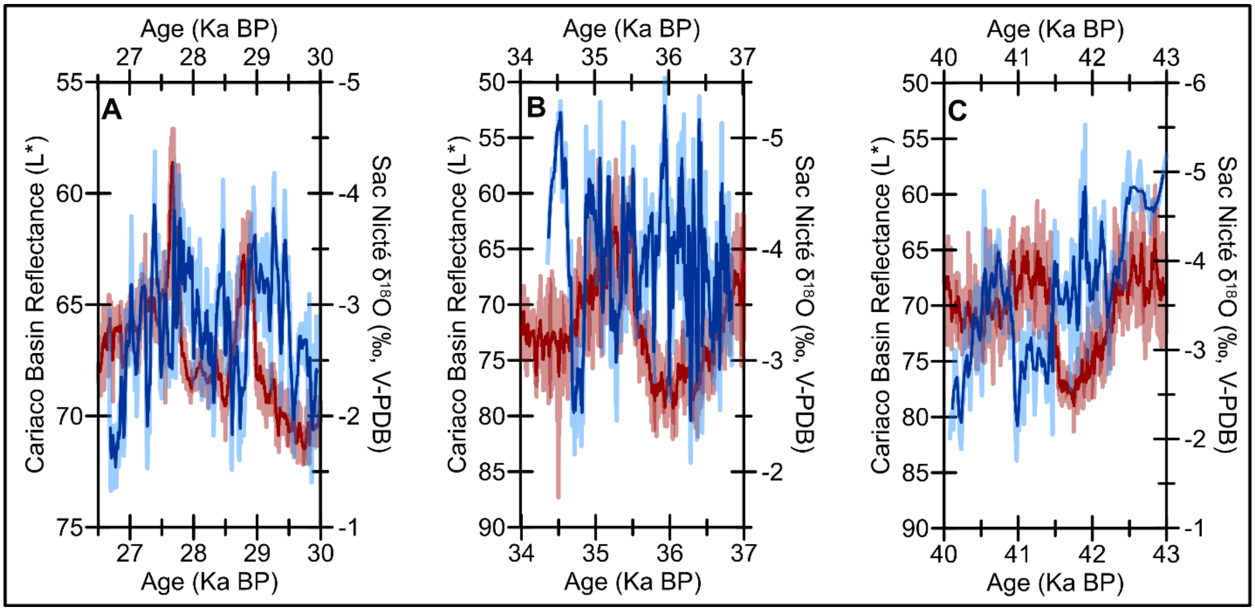

Figure S9. Sac Nicté oxygen isotope record compared with Cariaco Basin reflectance record[14] over (A) section 3, (B) section 2, and (C) section 1. Inconsistencies in correlation among these records are likely due to chronological uncertainties >200 years[14].

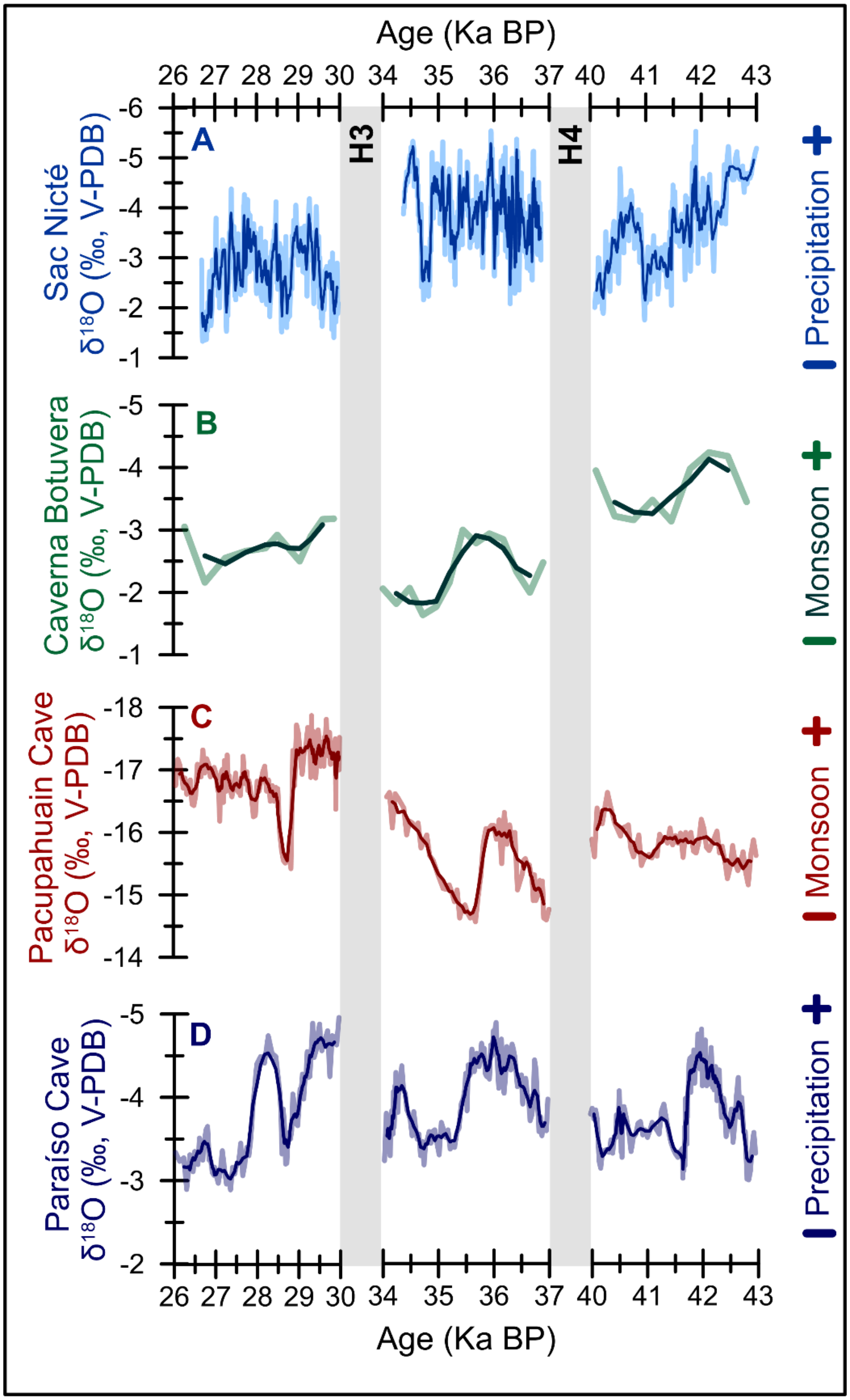

Figure S10. (A) Sac Nicté record compared with records from the Southern Hemisphere over the same time interval – (B) Brazilian cave oxygen isotope record[15], (C) Peruvian cave oxygen isotope record[16], and (D) Amazon basin cave oxygen isotope record[17]. Inconsistencies in the timing of Heinrich events among these records are likely due to reported chronological uncertainties of 0.4%[16], 0.5%[15], and 0.5%[17].

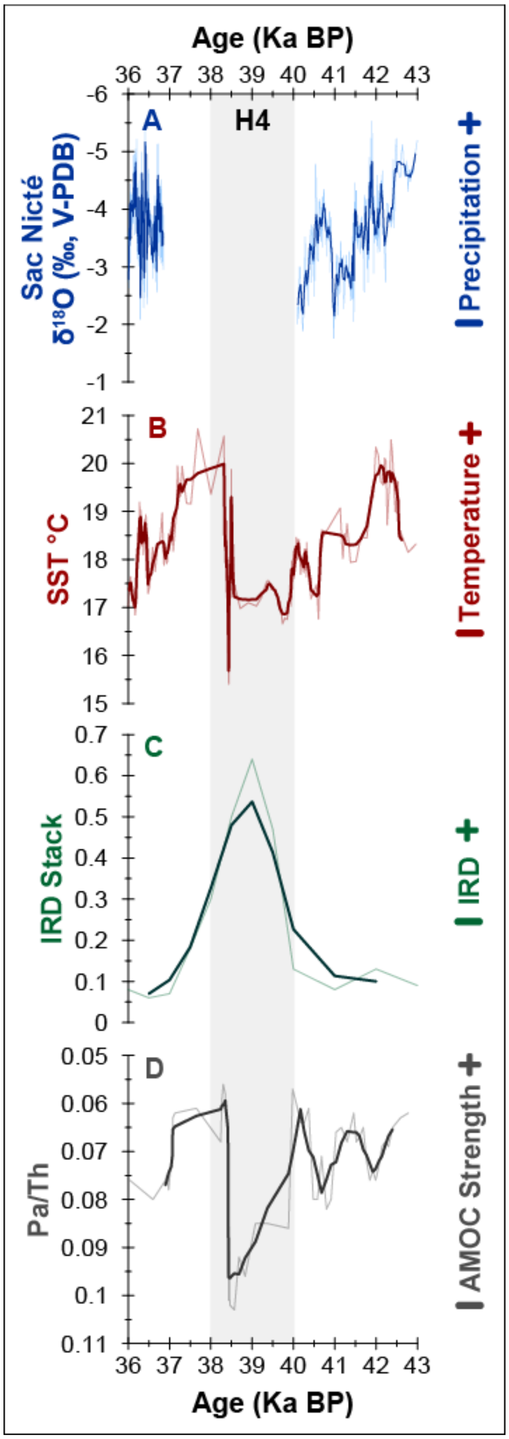

Figure S11. (A) Sac Nicté record compared with (B) Core MD95-2036 alkenone SST record from the Bermuda Rise[3], (C) ice-rafted debris (IRD) Stack[18], and (D) Pa/Th record (a record of AMOC strength)[19]. The comparison is enlarged to cover the interval spanning Heinrich 4. The mechanism for the observed millennial-scale precipitation variability recorded in Sac Nicté is SST changes due to AMOC strength fluctuations, controlled by North Atlantic freshwater forcing.

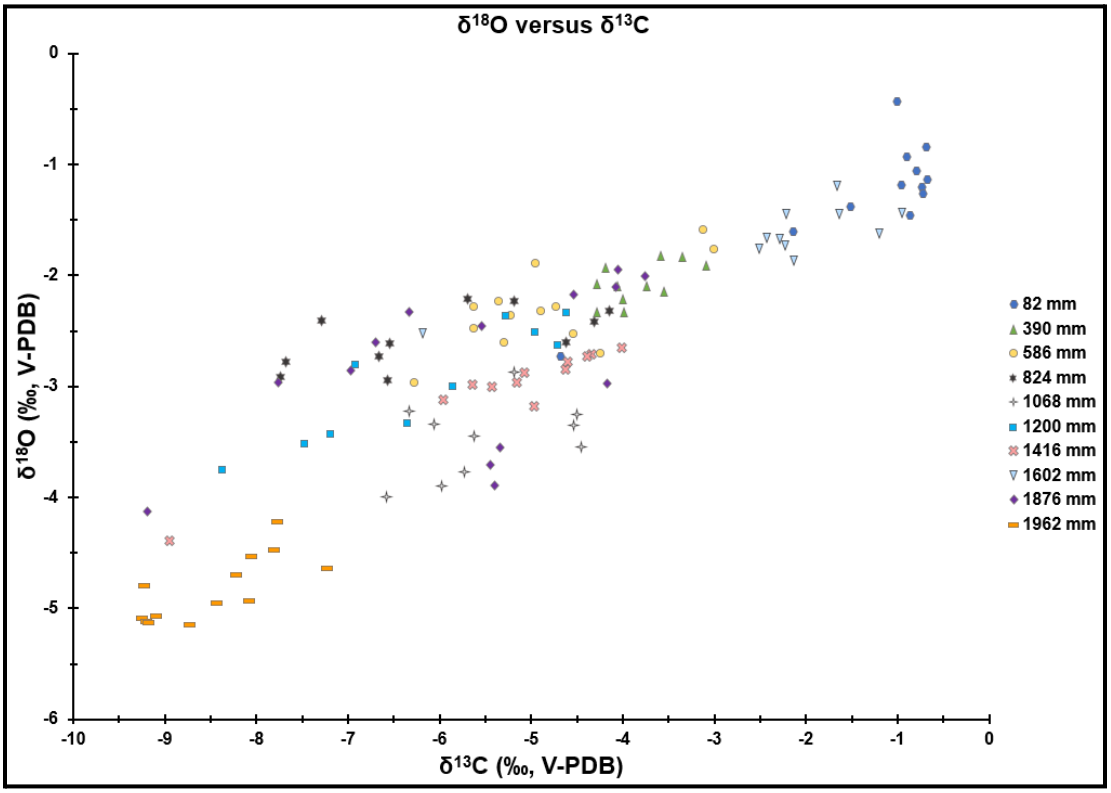

Figure S12. Hendy test[20] $\delta^{18}O$ versus $\delta^{13}C$ values for 10 growth profiles. In the RS cave system, precipitation is likely the primary driver of both $\delta^{18}O$ and $\delta^{13}C$ variability, causing the observed correlation (see Interpretation of Proxies section in main text and Fig. S3).

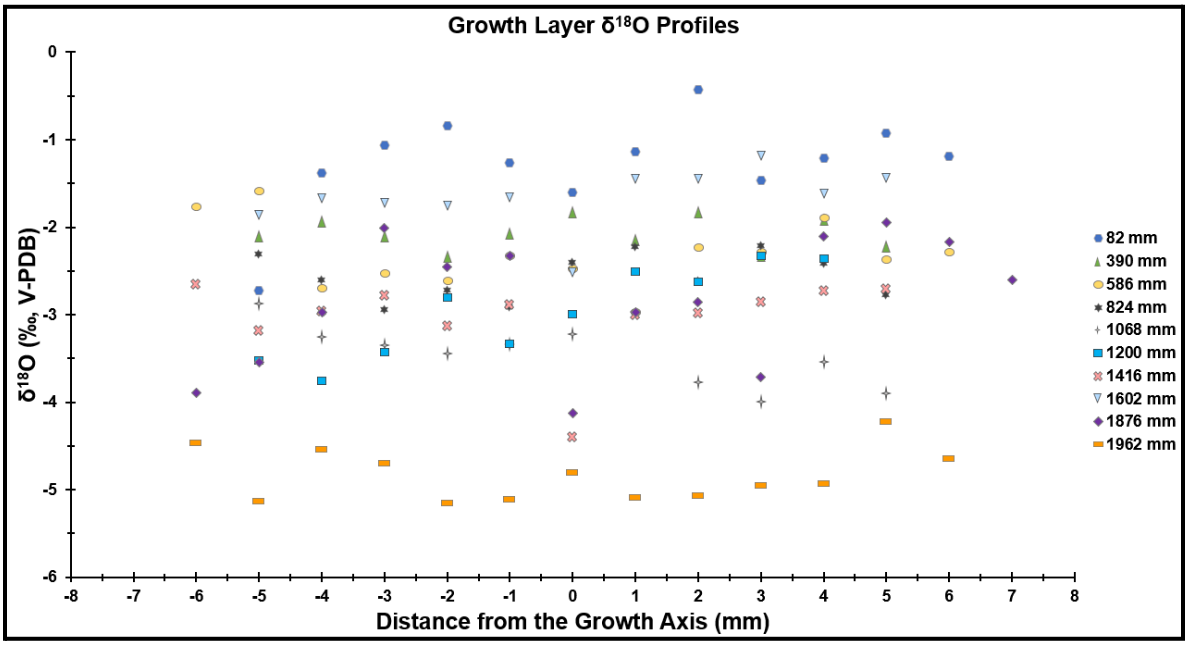

Figure S13. Hendy test[20] $\delta^{18}O$ values versus distance from growth axis for 10 growth profiles. Note $\delta^{18}O$ values remain relatively constant along each growth layer.

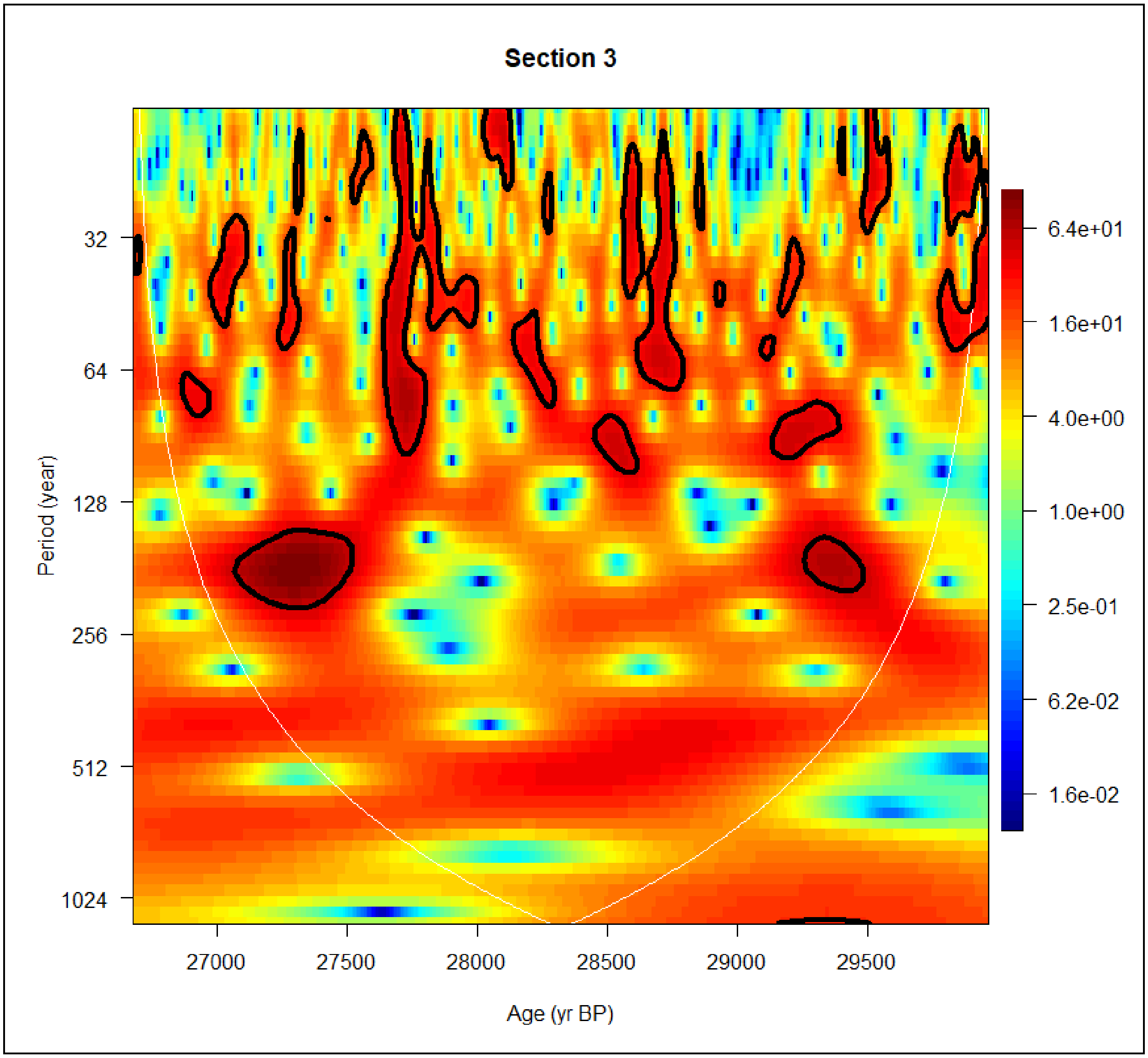

Figure S14. Wavelet spectral power analysis performed in R for section 3 $\delta^{18}O$ record (~26-30 ka BP) with scale of spectral power on right.

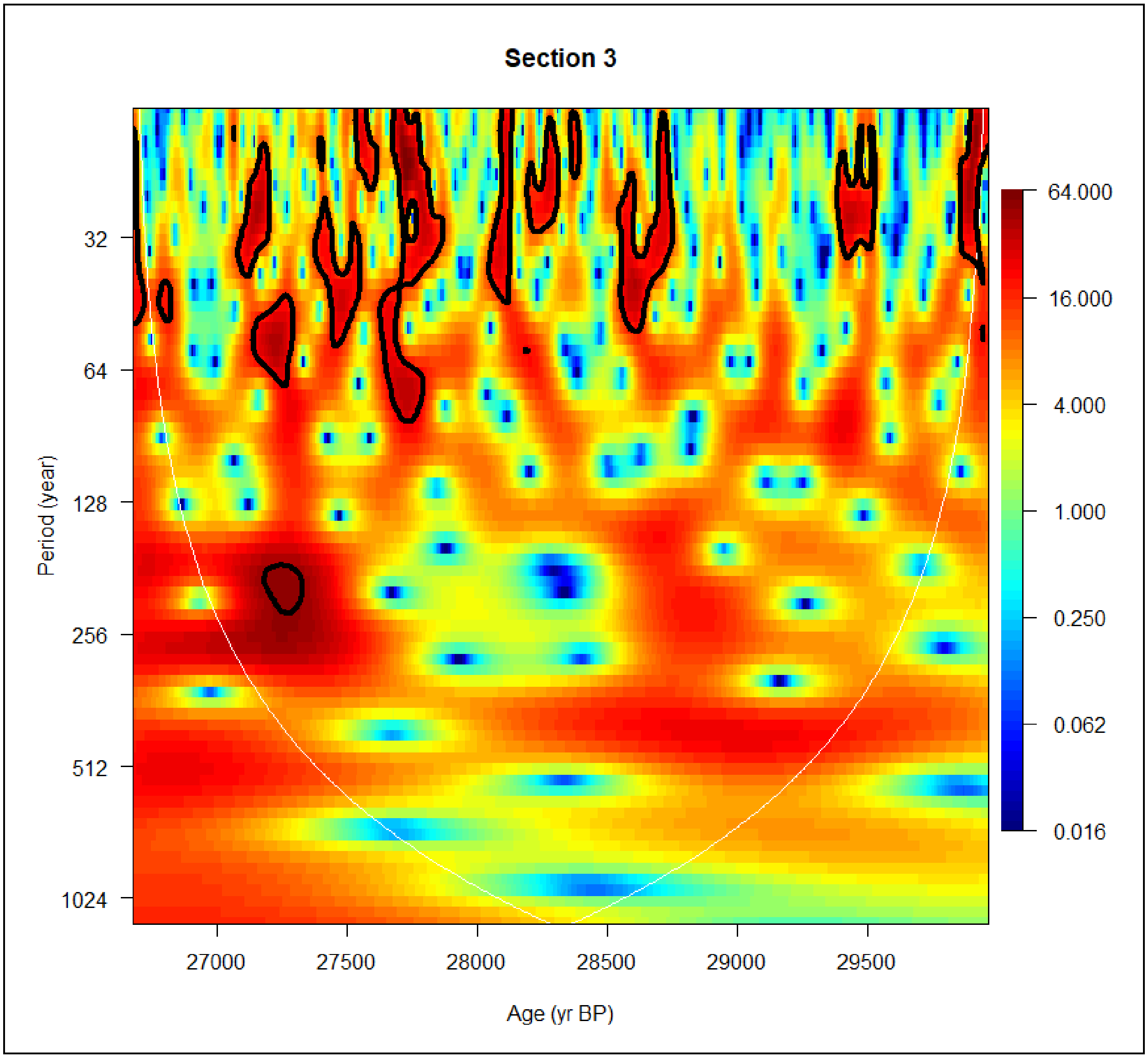

Figure S15. Wavelet spectral power analysis performed in R for section 3 $\delta^{13}C$ record (~26-30 ka BP) with scale of spectral power on right.

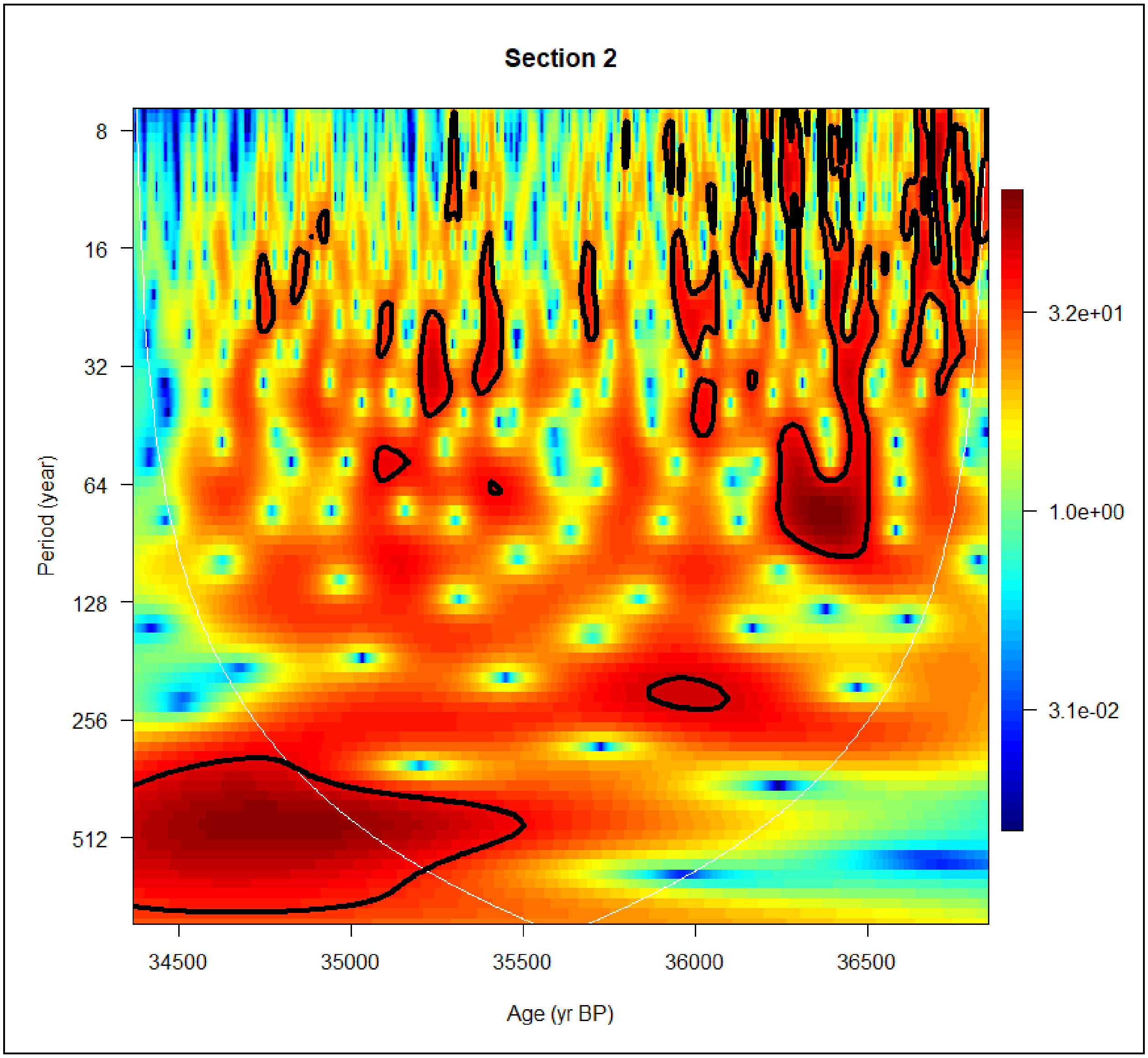

Figure S16. Wavelet spectral power analysis performed in R for section 2 $\delta^{18}O$ record (~34-37 ka BP) with scale of spectral power on right.

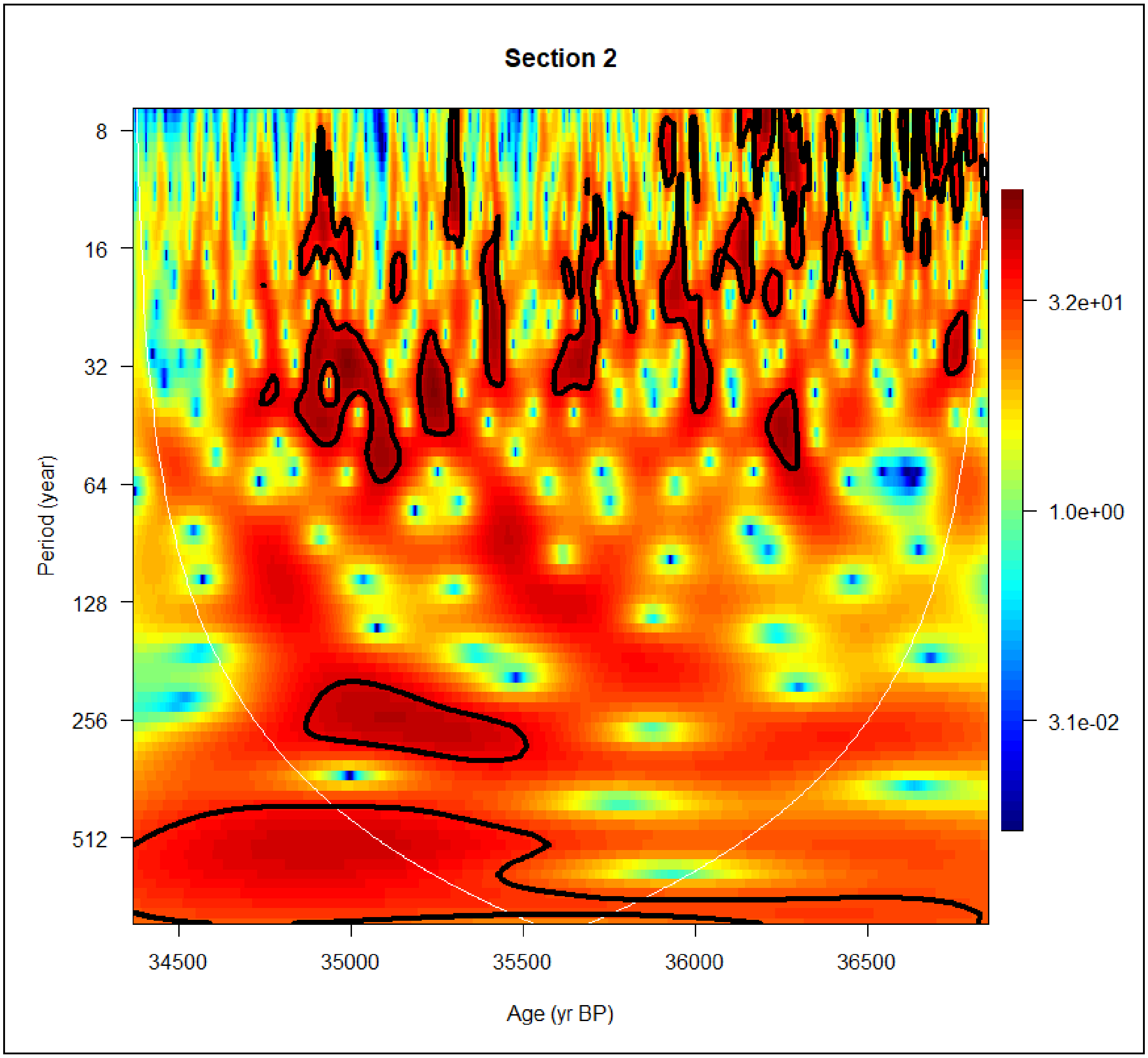

Figure S17. Wavelet spectral power analysis performed in R for section 2 $\delta^{13}C$ record (~34-37 ka BP) with scale of spectral power on right.

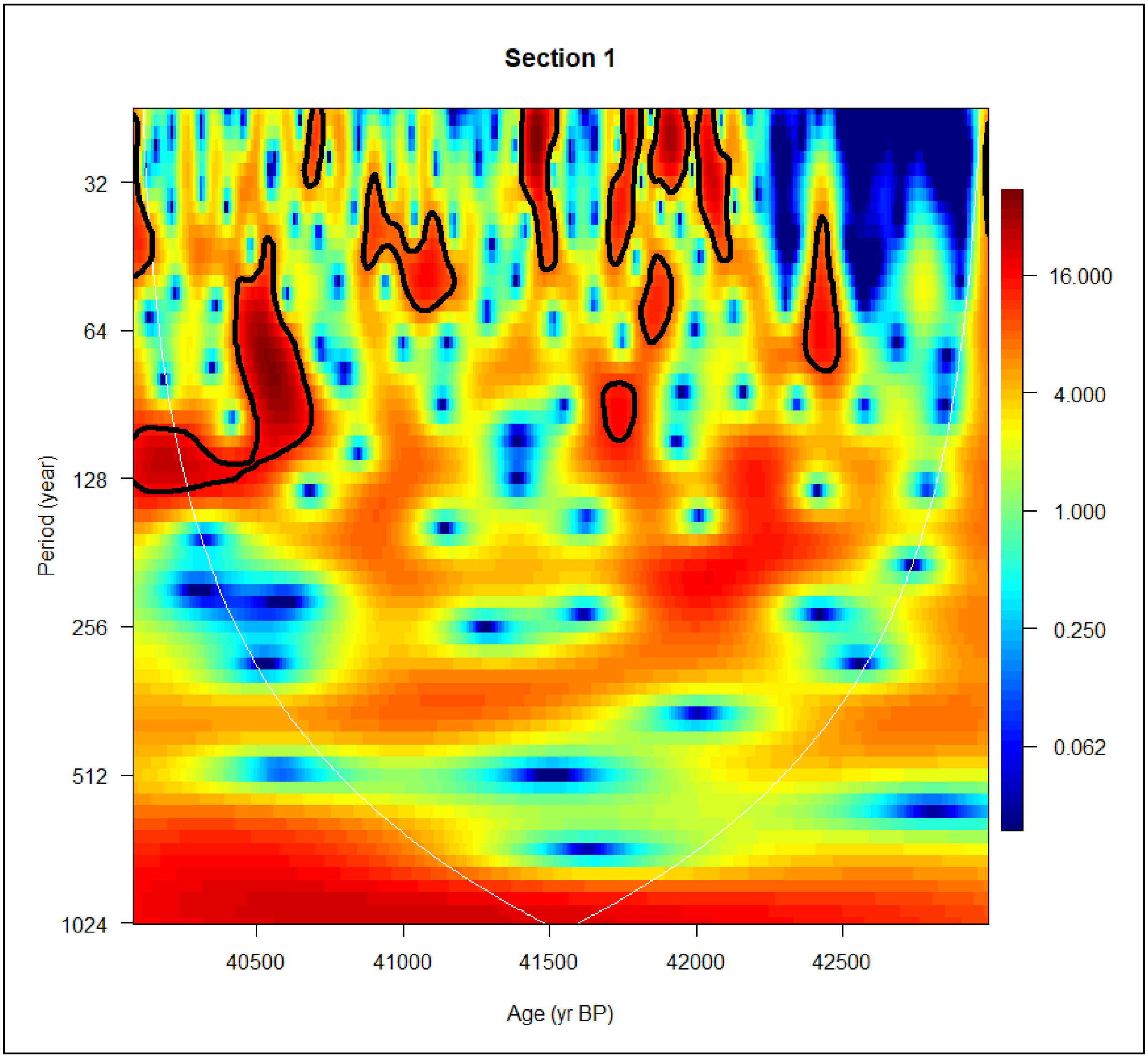

Figure S18. Wavelet spectral power analysis performed in R for section 1 $\delta^{18}O$ record (~40-43 ka BP) with scale of spectral power on right.

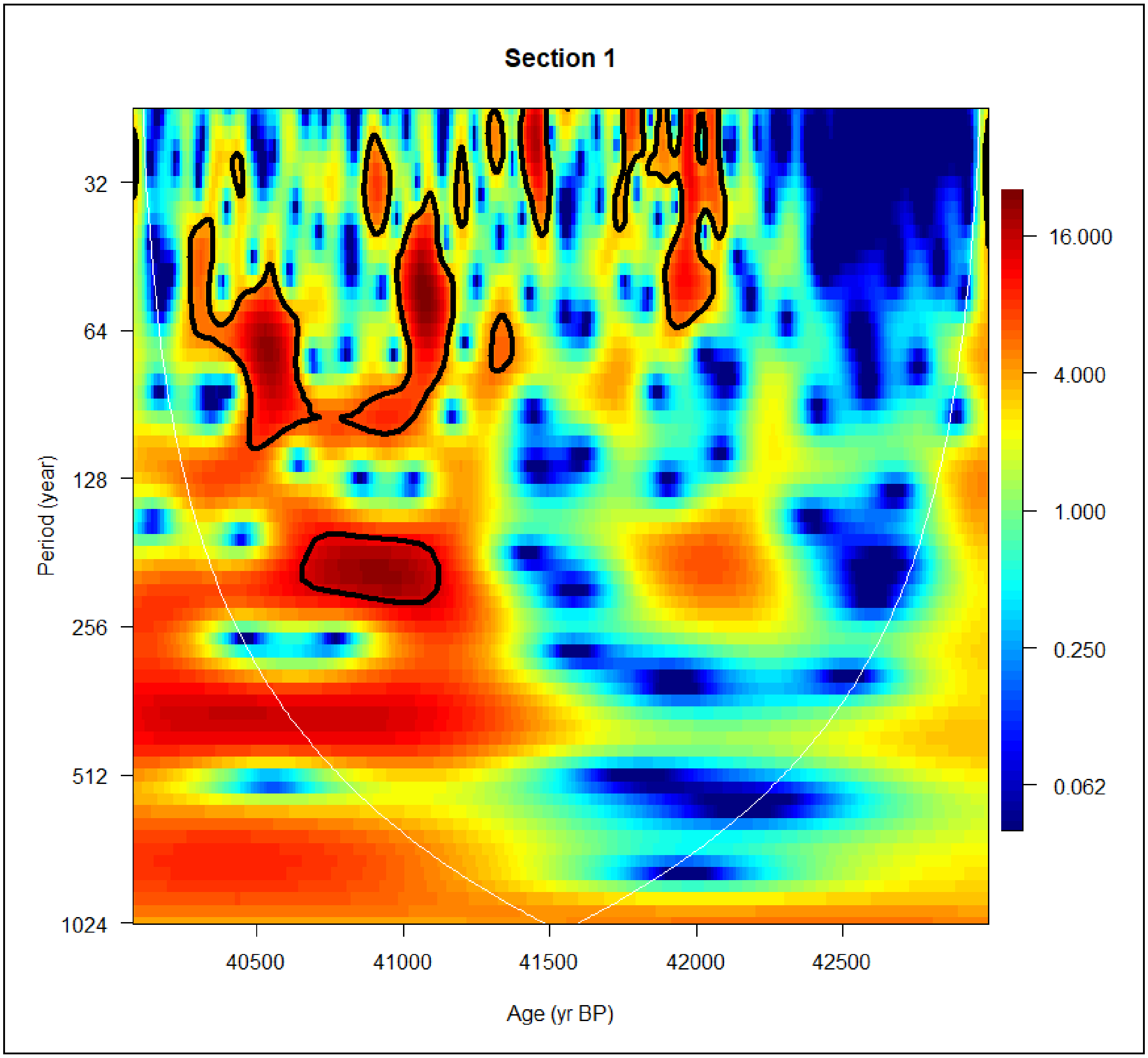

Figure S19. Wavelet spectral power analysis performed in R for section 1 $\delta^{13}C$ record (~40-43 ka BP) with scale of spectral power on right.

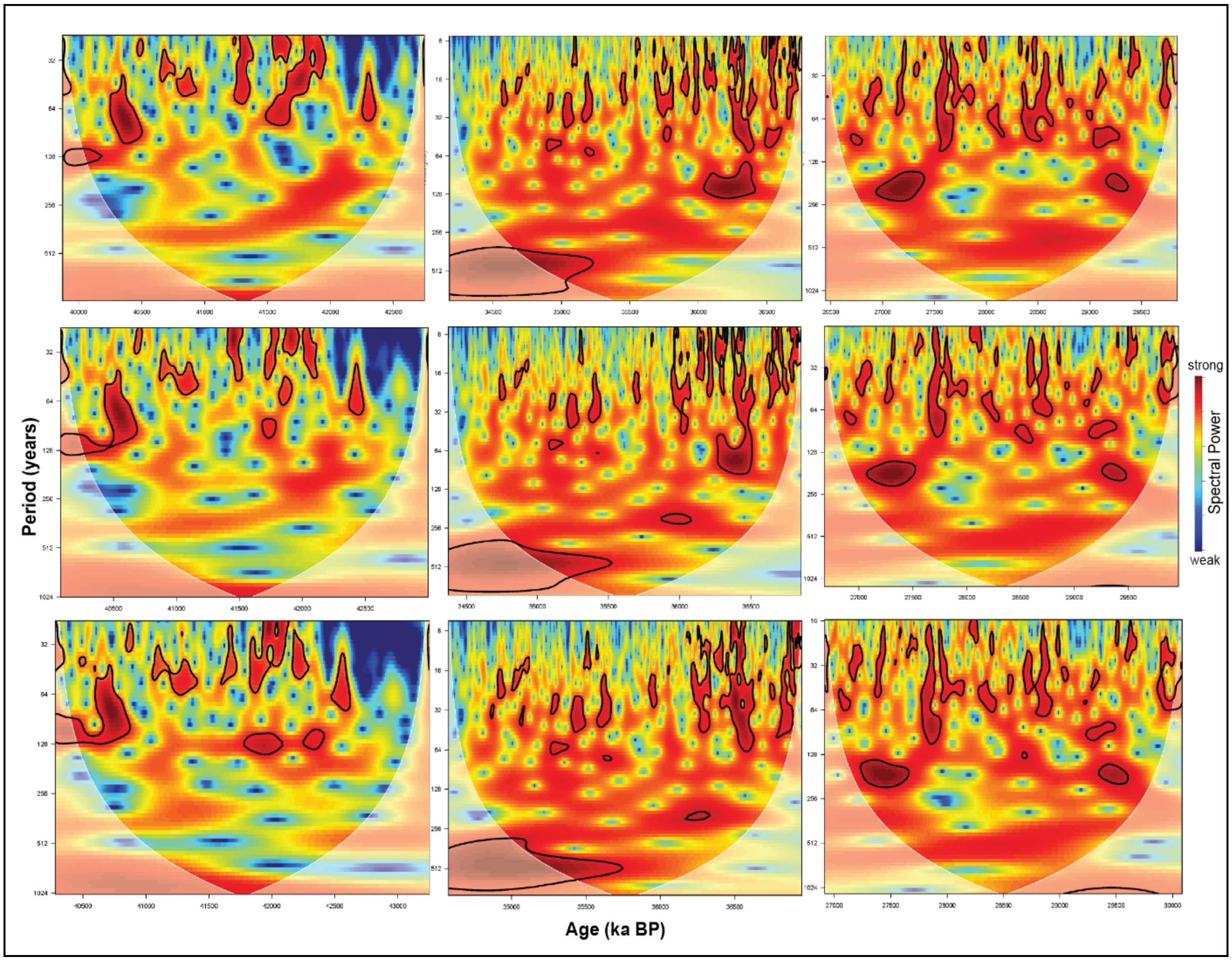

Figure S20. Wavelet spectral power analysis performed in R for on the two time series that represent the 95% confidence interval of the SN age model. The top row represents the youngest (low) simulation, the middle row is the mean simulation (SN age model), and the bottom row is the oldest (high) simulation, covering the extremes and the least likely curves to represent the oxygen isotope data. We obtained nearly identical significant power spectra for all three time series. Upper left = S1 low; center left = S1 mean; lower left = S1 high; upper middle = S2 low; center middle = S2 mean; lower middle = S2 high; upper right = S3 low; center right = S3 mean; lower right = S3 high.

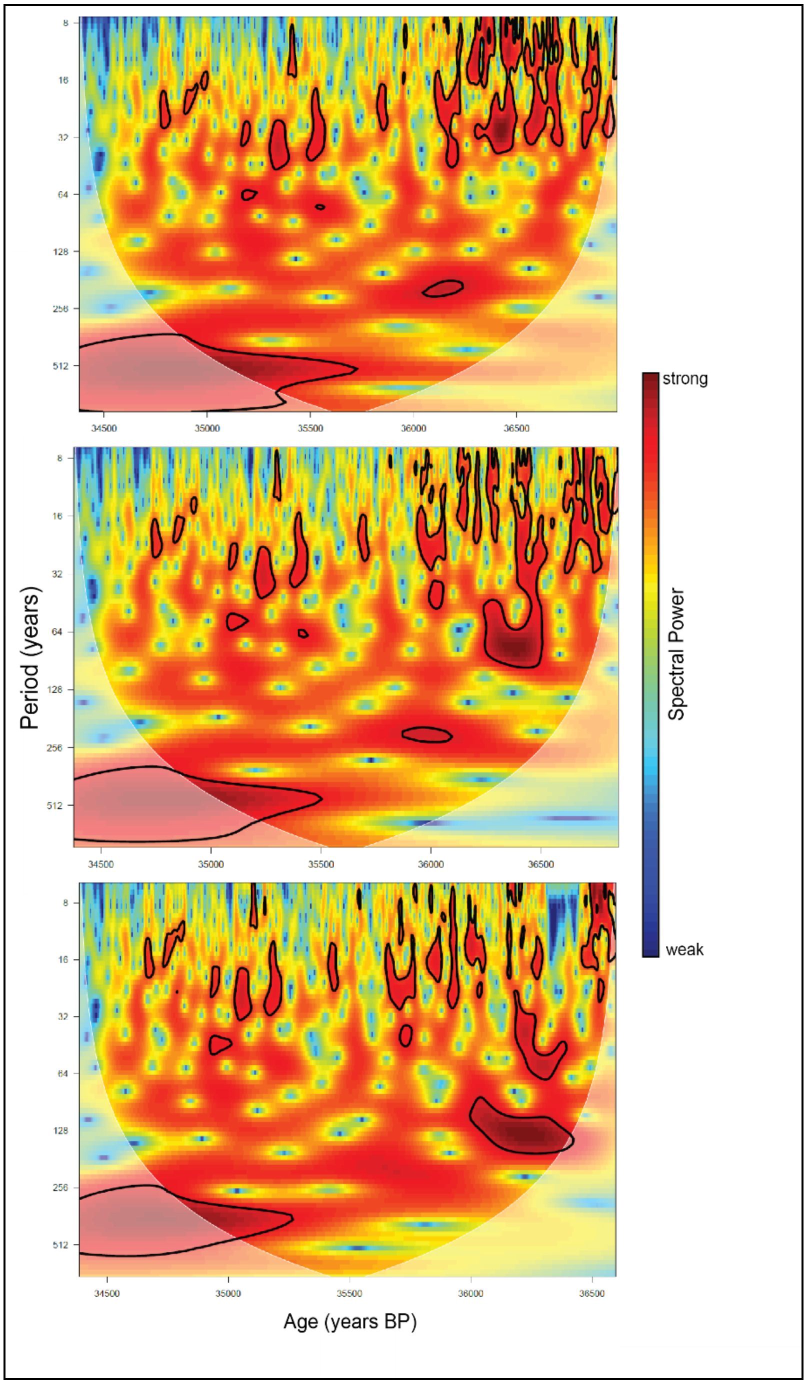

Figure S21. Wavelet spectral power analysis performed in R for section 2 $\delta^{18}O$ record with timescale expanded (top), normal (middle), and contracted (bottom) by the average age uncertainty for the section (±205 years). Note x-axis age between panels. Only the ages where higher resolution allows the observation of decadal variability (i.e., ~36.2 and ~36.7 ka BP) were expanded/contracted. All other ages in the section were not adjusted. New age models for the section were created for the expanded and contracted scenarios, followed by wavelet spectral power analysis for both.

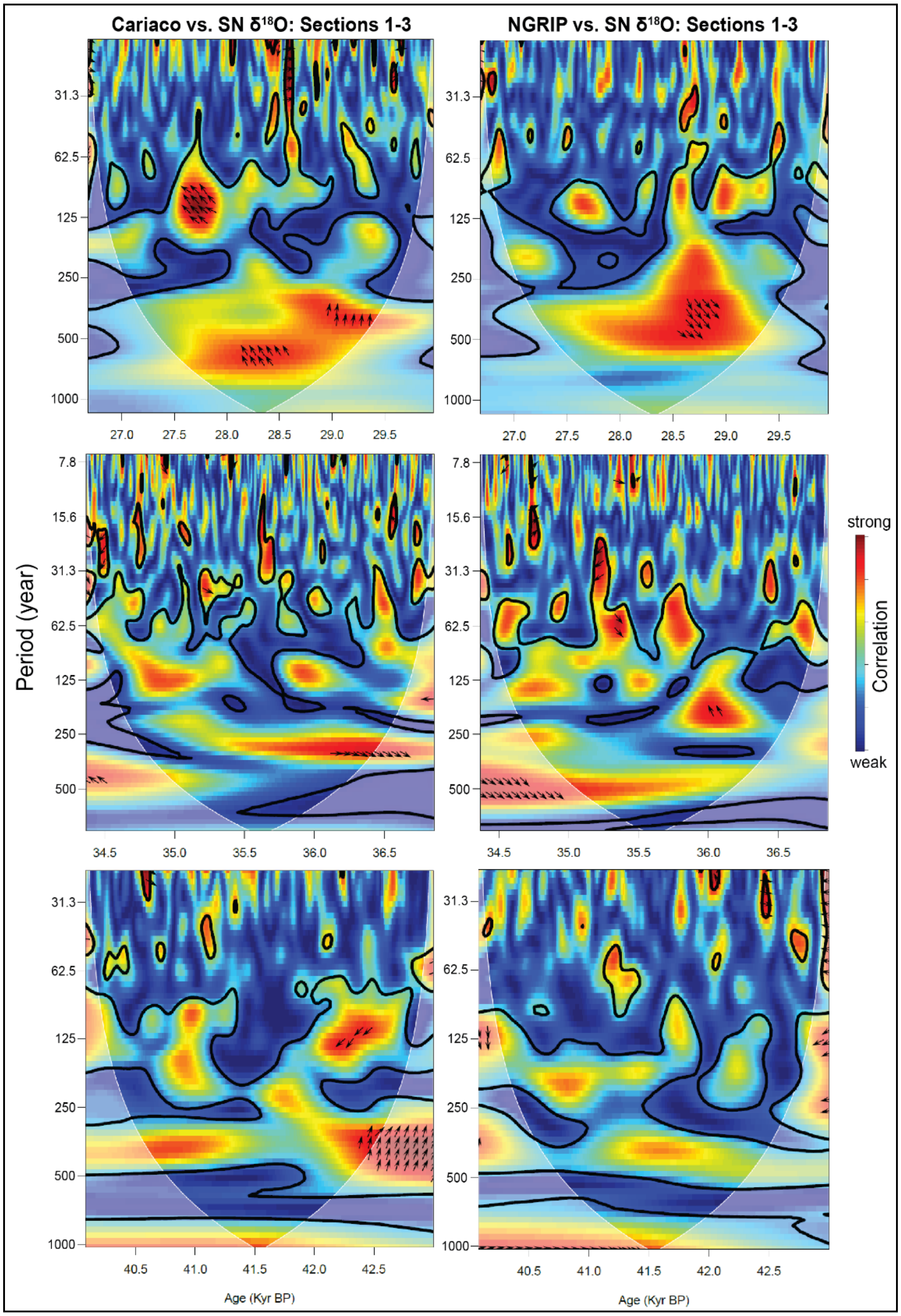

Figure S22. Wavelet correlation analysis performed in R for SN $\delta^{18}O$ record versus the Cariaco Basin reflectance[14] (left) and NGRIP[2] (right) records. Top row = section 3, middle row = section 2, and bottom row = section 1. Statistically significant high wavelet correlations are present for both records. Correlations were performed using the R package biwavelet[8-10]. Note the lack of arrows indicating phase differences is to be expected due to chronological uncertainties associated with the records (see Table S1).

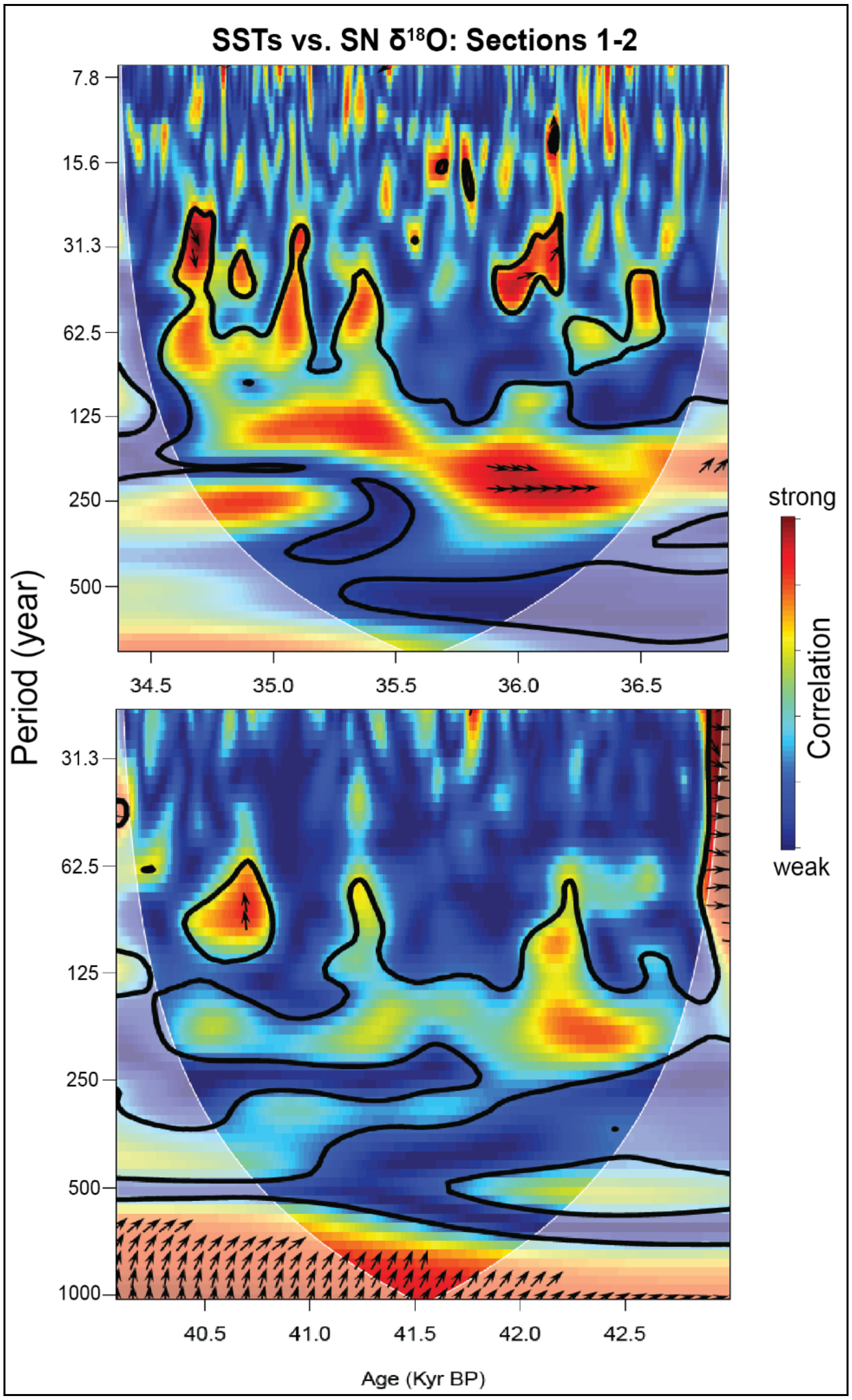

Figure S23. Wavelet correlation analysis performed in R for SN $\delta^{18}O$ record versus available alkenone temperature record from the Bermuda Rise[3]. Top row = section 2 and bottom row = section 1. In general, statistically significant high wavelet correlations are present. Correlations were performed using the R package biwavelet[8-10]. Note the lack of arrows indicating phase differences is to be expected due to chronological uncertainties associated with the records (see Table S2).

REFERENCES CITED

1 Andersen, K. K. *et al.* The Greenland Ice Core Chronology 2005, 15–42 ka. Part 1: constructing the time scale. *Quaternary Science Reviews* **25**, 3246-3257 (2005).

2 NGRIP members. High-resolution record of Northern Hemisphere climate extending into the last interglacial period. *Nature* **431**, 147-151 (2004).

3 Sachs, J. P. & Lehman, S. J. Subtropical North Atlantic temperatures 60,000 to 30,000 years ago. *Science* **286**, 756-759 (1999).

4 Cheng, H. *et al.* Improvements in 230Th dating, 230Th and 234U half-life values, and U-Th isotopic measurements by multi-collector inductively coupled plasma mass spectrometry. *Earth and Planetary Science Letters* **372**, 82-91 (2013).

5 Jaffey, A. H., Flynn, K. F., Glendenin, L. E., Bentley, C. & Essling, A. M. Precision Measurement of Half-Lives and Specific Activities of 235U and 238U. *Physical Review C* **4**, 1889-1906 (1971).

6 Breitenbach, S. F. M. *et al.* Constructing Proxy Records from Age models (COPRA). *Climate of the Past* **8**, 1765-1779 (2012).

7 Medina-Elizalde, M. *et al.* Synchronous precipitation reduction in the American Tropics associated with Heinrich 2. *Scientific Reports* **7**, 11216, doi:10.1038/s41598-017-11742-8 (2017).

8 Gouhier, T. C., Grinsted, A. & Simko, V. R package biwavelet: Conduct Univariate and Bivariate Wavelet Analyses (version 0.20.19). (2021).

9 Liu, Y., San Liang, X. & Weisberg, R. H. Rectification of the bias in the wavelet power spectrum. *Journal of Atmospheric and Oceanic Technology* **24**, 2093-2102 (2007).

10 Torrence, C. & Compo, G. P. A practical guide to wavelet analysis. *Bulletin of the American Meteorological Society* **79**, 61-78 (1998).

11 Hodell, D. A. *et al.* An 85-ka record of climate change in lowland Central America. *Quaternary Science Reviews* **27**, 1152-1165, doi:Doi 10.1016/J.Quascirev.2008.02.008 (2008).

12 Escobar, J. *et al.* A similar to 43-ka record of paleoenvironmental change in the Central American lowlands inferred from stable isotopes of lacustrine ostracods. *Quaternary Science Reviews* **37**, 92-104, doi:Doi 10.1016/J.Quascirev.2012.01.020 (2012).

13 Wang, Y. J. *et al.* A high-resolution absolute-dated Late Pleistocene monsoon record from Hulu Cave, China. *Science* **294**, 2345-2348, doi:Doi 10.1126/Science.1064618 (2001).

14 Deplazes, G. *et al.* Links between tropical rainfall and North Atlantic climate during the last glacial period. *Nature Geoscience* **6**, 213-217 (2013).

15 Wang, X. *et al.* Millennial-scale precipitation changes in southern Brazil over the past 90,000 years. *Geophys. Res. Lett.* **34**, L23701 (2007).

16 Kanner, C. L., Burns, J. S., Cheng, H. & Edwards, R. L. High-Latitude Forcing of the South American Summer Monsoon During the Last Glacial. *Science* **335**, 570-573 (2012).

17 Wang, X. *et al.* Hydroclimate changes across the Amazon lowlands over the past 45,000 years. *Nature* **541**, 204-207 (2017).

18 Lisiecki, L. E. & Stern, J. V. Regional and global benthic δ18O stacks for the last glacial cycle. *Paleoceanography* **31**, 1368-1394 (2016).

19 Henry, L. G. *et al.* North Atlantic Ocean circulation and abrupt climate change during the last glaciation. *Science* **353**, 470-474 (2016).

20 Hendy, C. H. The isotopic geochemistry of speleothems—I. The calculation of the effects of different modes of formation on the isotopic composition of speleothems and their applicability as palaeoclimatic indicators. *Geochimica Et Cosmochimica Acta* **35**, 801-824 (1971).
